# Evaluating the Antimicrobial Activity of Curcumin-loaded Lipid Nanocarriers When Applying Photodynamic and Sonodynamic Therapy for the Treatment of Skin Infections

**DOI:** 10.1007/s11095-026-04138-9

**Published:** 2026-10-07

**Authors:** Omar Molina-Alejandre, Angélica Villanueva-Martínez, María Guadalupe Nava-Arzaluz, Laura Abril Pérez-Carranza, Andrea Becerril-Osnaya, Gonzalo Hedain López-Mera, Raúl Dalí Cruz-Morales, Alma Alejandra Luna-Gómez, Doris Cerecedo, Ivette Martínez-Vieyra, María Andrea Trejo-Márquez, Selene Pascual-Bustamante, Yolanda I. Chirino, Octavio Ispanixtlahuatl-Meraz, Adriana Ganem-Rondero

**Affiliations:** 1 Laboratorio de Investigación y Posgrado en Tecnología Farmacéutica (L-322), Facultad de Estudios Superiores Cuautitlán, Universidad Nacional Autónoma de México, Estado de México, Av. 1o de Mayo s/n, Cuautitlán Izcalli, Estado de México C.P. 54740 México; 2 Bacteriología diagnóstica, Facultad de Estudios Superiores Cuautitlán, Universidad Nacional Autónoma de México, Av. 1o de Mayo s/n, Cuautitlán Izcalli, C.P. 54740 Estado de México México; 3 Departamento de Ingeniería, Facultad de Estudios Superiores Cuautitlán, Universidad Nacional Autónoma de México, Estado de México, Carr. Cuautitlán-Teoloyucan Km. 2.5, San Sebastián Xhala, Cuautitlán Izcalli, 54714 Estado de México Mexico; 4 Laboratorio de Hematobiología, Escuela Nacional de Medicina y Homeopatía, Instituto Politécnico Nacional, Guillermo Massieu Helguera, No. 239, Fracc. “La Escalera”, Ticomán, Gustavo a. Madero, C.P. 07320 Ciudad de México México; 5 Laboratorio de Postcosecha de Productos Vegetales, Centro de Asimilación Tecnológica, Facultad de Estudios Superiores Cuautitlán, Universidad Nacional Autónoma de México, Av. Jiménez Cantús/n, San Juan Atlámica, Cuautitlán Izcalli, C.P. 54729 Estado de México Mexico; 6 Laboratorio 10, Unidad de Biomedicina, Facultad de Estudios Superiores Iztacala, Universidad Nacional Autónoma de México, Avenida de los Barrios Número 1, Colonia Los Reyes Iztacala, Tlalnepantla, C.P. 54090 Estado de México México

**Keywords:** antimicrobial activity, curcumin, lipid nanocarriers, photodynamic therapy, sonodynamic therapy

## Abstract

**Purpose:**

The antimicrobial activity of curcumin is the focus of this article, which sought to enhance it by formulating curcumin in lipid nanocarriers, and by applying Sonodynamic Therapy (ST) and Photodynamic Therapy (PT).

**Methodology:**

Curcumin-loaded lipid nanocarriers were formulated and characterized for size, zeta potential, morphology, FTIR, and DSC. The performance of the formulation was evaluated using antioxidant activity assays, *in vitro* release assays, occlusion assays, and stability assays under various conditions. The distribution of curcumin in pig skin *in vitro* was assessed using permeability assays and visualized by confocal microscopy. The antimicrobial activity was assessed against Methicillin-resistant *Staphylococcus aureus* (MRSA) and *Pseudomonas aeruginosa,* applying PT or ST under different conditions. The cytotoxicity of the formulation was evaluated using HaCaT cells.

**Results and discussion:**

The curcumin-loaded lipid nanocarriers had a size of 63 nm, a polydispersity index of 0.202, a zeta potential of -41 mV, and an encapsulation efficiency > 95%. The properties of curcumin were enhanced by the lipid nanocarrier formulation, including stability, antioxidant activity, and skin-penetrating capacity. The antimicrobial activity of the formulation against the two strains was very low when combined with PT (< 12%), whereas ST achieved complete inhibition of *P. aeruginosa*. HaCaT viability was unaffected by the formulation or light.

**Conclusions:**

The proposed formulation may be a promising alternative for improving curcumin properties, preserving its stability, and enhancing its antimicrobial activity, particularly when combined with ST.

**Graphical Abstract:**

**Figure:**
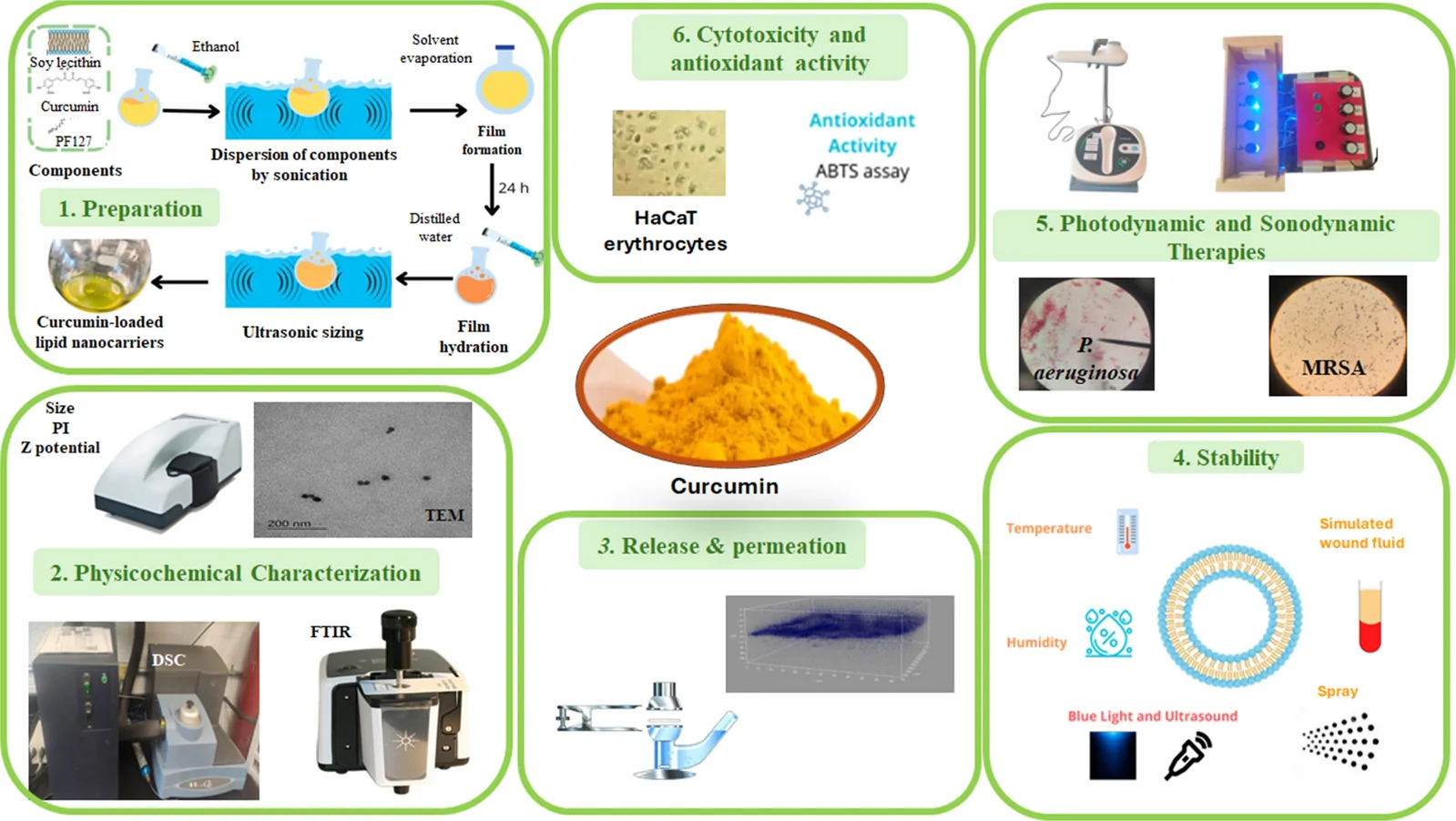

**Supplementary Information:**

The online version contains supplementary material available at https://doi.org/10.1007/s11095-026-04138-9.

## Introduction

Curcumin is a molecule extracted from the roots of *Curcuma longa L*. (turmeric). It is a curcuminoid, a low-molecular-weight polyphenol with hydrophobic properties and a distinctive yellow color. Its molecular structure can exist in two tautomeric forms: keto and enolate. Curcumin is a pleiotropic molecule with proven antioxidant, anti-inflammatory, hypoglycemic, wound healing, and antitumor properties, as well as being able to induce apoptosis in various cellular systems. The safety and lack of toxicity of curcumin, even at high doses, are supported by clinical trials [1–3]. Furthermore, curcumin acts as a photosensitizer (PS) and exhibits bactericidal effects under blue-light irradiation [4]. It can also enhance antibacterial properties when combined with other bacteriostatic substances. Additionally, it has been suggested that low-intensity ultrasound can activate curcumin, thereby stimulating the production of Reactive Oxygen Species (ROS). Therefore, curcumin has been recognized as a relatively new PS and/or Sonosensitizer (SS), offering great potential as an antimicrobial agent, provided that a suitable formulation must be developed for its application, given its high lipophilicity, which limits its use [5–8].

Antimicrobial resistance and tolerance (AMR&T) occur when microorganisms, such as bacteria, viruses, fungi, or parasites, develop mechanisms to evade the effects of drugs [9]. The continuous use of antimicrobials has contributed to the emergence of AMR&T, and factors such as mutation, spontaneous evolution, and gene transfer are key determinants in this process [10, 11]. To address AMR&T, actions such as improving surveillance, optimizing antibiotic use, developing new antimicrobial agents, promoting vaccination, raising public awareness, and fostering international cooperation are required [12]. In addition, there is an urgent need to implement advanced drug-delivery strategies, in which scientists play a crucial role in combating AMR&T and future prevention by designing innovative systems that utilize both conventional and novel anti-infectives [13].

Both Photodynamic Therapy (PT) and Sonodynamic Therapy (ST) have emerged as new alternatives to combat AMR&T. Both treatments involve the application of a PS or SS, using a radiation source (visible light or ultrasound) capable of exciting the molecule to produce singlet oxygen (^1^O_2_), superoxide anions (O_2_^−^), and hydroxyl radicals (OH), generated by the transfer of energy from the PS or SS in the triplet state to endogenous molecular oxygen. Although the main advantage of ST over PT is the greater penetration of ultrasound through tissue than of light, the penetration of PS and SS molecules is poor, reducing the efficiency of both therapies. Although both therapies are innovative, they are limited to local or topical use for treating skin infections [5, 14].

Advances in nanotechnology have enabled the development of nanoscale delivery systems that improve drug penetration and distribution in the skin. In this sense, lipid nanocarriers are recognized not only for their ability to encapsulate both hydrophilic and lipophilic drugs, but also for their biocompatibility, biodegradability, and well-tolerated topical application [15, 16].

For the above reasons, this work focused on developing, characterizing, and evaluating curcumin-loaded lipid nanocarriers, and assessing their potential as antimicrobial agents in combination with PT or ST against two clinical strains commonly found in skin infections: Methicillin-resistant *Staphylococcus aureus* (MRSA) and *Pseudomonas aeruginosa*.

## Materials and Methods

### Materials

Curcumin (70%), Pluronic® F-127 (PF127), dimethyl sulfoxide (DMSO), methylthiazolyldiphenyl-tetrazolium bromide (MTT), 2,2'-azino-bis(3-ethylbenzothiazoline-6-sulfonic acid (ABTS), potassium persulfate, 6-hydroxy-2,5,7,8-tetramethylchroman-2-carboxylic acid (Trolox), rhodamine conjugated with phalloidin, Iscove′s modified Dulbecco′s medium (IMDM), and Triton® X-100 were all obtained from Sigma-Aldrich®, USA. Hydrochloric acid (HCl), absolute ethanol, and isopropanol were purchased from Meyer®, Mexico. Standard Soy Lecithin (SSL, composed, in order of abundance, of phosphatidylcholine, phosphatidylethanolamine, phosphatidylinositol, and triglycerides) and water-dispersible lecithin (WSL, fortified with solubilizing agents such as tertiary amine oxides, alkyl alcohol sulfate salts, alkylamidopropyl betaines, sodium alkenesulfonates, alkyl sulfoacetates, and sulfosuccinate monoalkyl amide disodium salt to increase its aqueous dispersibility) were obtained from INTERQUIMICA, Mexico. Pluronic® P-123 (PP123) was purchased from BASF®, USA. Sodium hydroxide ≥ 99.0%, sodium chloride ≥ 99.0%, and monobasic sodium phosphate ≥ 99.0% were acquired from J.T. Baker®, Mexico. Standard Methods Agar was provided by DIBICO®, Mexico. StratM® membranes were purchased from Merck Millipore®, Germany. All the reagents were of analytical grade and were used without further purification. Distilled and deionized water was obtained from a Milli-Q system (Millipore® Corp., MA, USA).

### Preparation of Curcumin-loaded Lipid Nanocarriers

To determine the optimal conditions for obtaining curcumin-loaded lipid nanocarriers, preliminary formulations were prepared through a 2^4^ factorial design, accounting for the following factors: phospholipid source (standard lecithin (SSL) or water-dispersible lecithin (WSL)), stabilizer (Pluronic® F127 or Pluronic® P123), type of solvent (ethanol or isopropanol), and curcumin concentration (0.16 or 0.5 mg/mL). Lipid nanocarriers were obtained using the film hydration method. Briefly, about 172 mg of soy lecithin (standard or lecithin easily dispersible in water), 28 mg of stabilizer and a known amount of curcumin were weighed into a 250 mL round-bottom flask. Then, 50 mL of ethanol or isopropanol was added, and the mixture was sonicated for 5 min. The solvent was evaporated under reduced pressure in a rotary evaporator (Buchi® R-3 HB, Switzerland) to form a film on the flask walls, and the flask was then dried in a desiccator for 24 h. Finally, the film was hydrated with 6 mL of distilled water and sonicated for 30 min at 40 kHz (Cole-Palmer®, model:08895–05, China).

### Characterization of Curcumin-loaded Lipid Nanocarriers

#### Particle Size, Polydispersity Index, Zeta Potential, and Morphology

Particle size and Polydispersity Index (PI) were determined by dynamic light scattering using a Zetasizer Nano-ZS90 from Malvern Instruments® (Worcestershire, West Midlands, United Kingdom) at 25°C and an angle of 90°. Measurements were performed in triplicate. The zeta potential was determined using the same equipment by electrophoretic light scattering. For this purpose, the samples were diluted with deionized water. The diluted sample had a pH of 6. The viscosity, refractive index, and dielectric constant of water at 25°C were accounted for when analyzing the samples in quintuplicate. For TEM measurements, 10 µL of curcumin-loaded lipid nanocarriers were placed on a TEM copper grid coated with a holey carbon film. A drop of 2% (w/v) phosphotungstic acid solution was placed on the TEM grid and allowed to interact with the sample for 10 s. Excess solution was carefully removed by touching the edge of the grid with filter paper. Subsequently, a drop of distilled water was placed on the grid and removed in the same manner to remove residual stain. Images were obtained using a JEOL® ARM-200F microscope at 200 kV with a low electron dose.

#### Entrapment Efficiency and Loading Capacity

Curcumin-loaded lipid nanocarriers were centrifuged at 13520 rcf for 20 min at 10°C. The pellet was separated from the supernatant and washed three times with distilled water. Subsequently, the pellet was resuspended in a mixture of ethanol:water (1:1). Quantification was performed by a previously validated UV/Vis spectrophotometric method (see Table S1, Supplementary material). The entrapment efficiency (EE) was calculated with Eq. 1.

1 $$EE=\frac{CUR~initial~amount - Free~CUR}{CUR~initial~amount}*100$$

where *CUR initial amount* is the amount of curcumin added to prepare the formulation, and *Free CUR* is the amount of free curcumin (determined in the pellet).

Loading Capacity (LC) was determined with Eq. 2, where *Encapsulated CUR* is the amount of curcumin loaded in lipid nanocarriers and *Total solids* is the total weight of the formulation components.

2 $$LC=\frac{Encapsulated~CUR}{Total~solids}$$

#### Attenuated Total Reflectance-Fourier Transform Infrared (ATR-FTIR) Spectroscopy and Differential Scanning Calorimetry

Samples of the raw materials, physical mixtures, and lipid nanocarriers were placed on a horizontal diamond ATR cell of an FTIR Cary 630 instrument (Agilent Technologies®, USA) to record their IR spectra. Measurements averaged 150 scans with a resolution of 16 cm^−1^. The thermal properties of the samples were determined using a Q20 Differential Scanning Calorimeter (TA Instruments®, USA). Approximately 2 mg of each sample was placed in sealed tzero aluminum cells and heated over the temperature range 20–280°C. Nitrogen was used as the transfer gas at a flow rate of 50 mL/min. For all determinations, the heating rate was 5°C/min, and an empty cell served as the reference.

### *In Vitro* Release Test

The *in vitro* release rate of curcumin from lipid nanocarriers was determined using Franz-type cells. A volume of the lipid nanocarrier dispersion containing 25 µg of curcumin was deposited into the donor compartment, while the receptor medium consisted of 1.5 mL of an ethanol:water (1:1) mixture, maintained at 34°C under constant stirring. A dialysis membrane with a molecular weight cutoff of 6–8 kDa (dialysis tube, Spectrum Laboratories, Inc., Switzerland) was placed between the donor and receptor compartments. The entire receptor medium was replaced hourly with fresh medium, and the amount of curcumin released was determined spectrophotometrically. For comparison, the same procedure was followed with placebo lipid nanocarriers and with a curcumin solution in ethanol:water (1:1). The release test was conducted in triplicate.

### *In Vitro* Permeation Test and Distribution in the Skin

The *in vitro* permeation test was also conducted using Franz-type cells, following the methodology described for the *in vitro* release study, except that pig ear skin (1.185 ± 0.1125 mm thickness) was placed between the donor and receptor compartments instead of a dialysis membrane. Permeation studies were conducted on both intact skin and skin from which the stratum corneum was removed by tape stripping, with 15 strips (hereafter referred to as wounded skin). The quantification of permeated curcumin was performed using a spectrophotometric method. Each permeation study was conducted in sextuplicate.

At the end of the permeation test, the skin was recovered to determine (i) the amount of curcumin remaining in the skin; and (ii) its distribution in the skin by confocal microscopy. The amount of curcumin remaining in the skin was determined as follows: Briefly, the skin was rinsed 15 times with distilled water to remove excess formulation. Subsequently, the skin was cut into thin portions, stirring for 24 h in 4 mL of ethanol:water (1:1). The solution was decanted, adjusting the volume to 5 mL with ethanol:water (1:1). Lastly, it was filtered through a 0.45 µm membrane, determining the amount of curcumin recovered by a spectrophotometric method. Each experiment was conducted in triplicate.

To evaluate the distribution of curcumin within the skin after the permeation experiments, the excised tissue was gently rinsed with PBS, fixed in 4% paraformaldehyde for 20 min at room temperature, and then washed three times in PBS (5 min each). Actin filaments were stained to visualize the cellular architecture using rhodamine-conjugated phalloidin (R415, Thermo Fisher Scientific®), diluted 1:1000 in PBS, incubating the samples at 37°C for 1.5 h, then washed 3 times with PBS (5 min each). The tissue sections were mounted in 0.9% saline–glycerol solution (v/v), and images were acquired using a Leica® TCS SP8 confocal microscope equipped with a 20 ×/0.75 NA objective. The pinhole was set to 1 Airy Unit, corresponding to a physical diameter of 56.6 µm for this optical configuration. Curcumin was visualized by its intrinsic autofluorescence at an excitation wavelength of 425 nm and emission collected between 500–550 nm. Rhodamine-phalloidin was excited at 540 nm, and emission was collected between 565 and 620 nm. Z-stack images were acquired from the basal to the apical region of the skin, covering a total depth of 150 µm with 24 optical sections (z-step of 6–7 µm), and three-dimensional reconstructions were generated using LAS X software (Leica Microsystems®). This imaging strategy enabled the visualization of curcumin’s spatial distribution within the skin.

### Transepidermal Water Loss (TEWL) and *in Vitro* Occlusion Test

The systems (pig ear skin mounted on Franz-type Cells) were equilibrated for 1 h using an aqueous solution as the receptor medium, and TEWL was measured with a Tewameter TM 300 (CK electronic GmbH®, Germany). Subsequently, the skin was treated with 50 µL of curcumin-loaded lipid nanocarriers. After 1 h of contact, the formulation was removed, and 30 min were allowed to elapse before TEWL was measured again. TEWL measurements were conducted within an acrylic cabinet, where humidity and temperature were continuously monitored. This study was carried out in sextuplicate.

According to the methodology previously reported [17], bakers (at a constant weight) were filled with 40 mL of distilled water and covered with Whatman Grade 42 filter paper (Sigma-Aldrich®, USA). A volume of 150 µL of lipid nanocarriers was spread on the surface of the filter paper. Water was used as a reference instead of lipid nanocarriers. Subsequently, the bakers were stored at 34°C and weighed at 0, 8, 24, and 48 h to calculate water loss through the filter paper. The occlusion factor (F) was calculated at 8, 24, and 48 h using Eq. (3).

3 $$F=\frac{ R-S}{R}*100$$

where *R* is the reference (water), and *S* is the sample (lipid nanocarriers). An *F* = 0 means no occlusive effect, while an *F* = 100 implies maximum occlusion. Each experiment was performed in triplicate.

### Stability Studies

#### Long-term and Accelerated Stability

The stability of the lipid nanocarrier suspension was determined in accordance with the conditions stipulated in the Mexican Official Standard NOM-073-SSA1-2015 [18] and the ICH Q1 Guideline on stability testing of drug substances and drug products [19]. Particle size, PI, Zeta Potential, and encapsulation of curcumin were observed under long-term stability conditions (5°C ± 3°C for 12 months) and accelerated stability conditions (25°C ± 2°C and 60% Relative Humidity, for 3 and 6 months).

#### Stability with Simulated Wound Fluid

The lipid nanocarrier dispersion (2 mL) was exposed to 0.6 mL of blood plasma (used as a simulated wound fluid, SWF) for 24 h at 34°C. Particle size and PI were examined according to the procedure described by Bernal-Chávez *et al*. [20].

#### Photostability and Sonostability

Lipid nanocarriers were irradiated with blue light (515 nm) and ultrasound waves (1 MHz, 3.5 W/cm^2^) for 36 min. In each experiment, the particle size, PI, Z potential, and curcumin encapsulation were monitored. Each analysis was performed in triplicate.

#### Stability After Atomization

Since lipid nanocarrier dispersion is intended for administration as a spray, it was of interest to investigate whether particle size and PI change. To accomplish this, the formulation was diluted tenfold in sterile physiological saline and atomized onto a surface area of 19.64 cm^2^ using a conventional mechanical atomizer. The parameters mentioned above were measured in the recovered fluid. The analysis was conducted three times for each sample.

### Antioxidant Activity

This test was performed using the ABTS^+^ color assay, as described by Re *et al*. [21], with some modifications. In brief, a 7 mM ABTS (2,2′-azino-bis(3-ethylbenzothiazoline-6-sulfonate)) solution was allowed to react with 5 mL of an aqueous K_2_S_2_O_8_ solution (2.45 mM) for 16 h at room temperature, protected from light. Next, 1 mL of the color assay was diluted to 100 mL with phosphate buffer (pH 7.42). This solution was read at 734 nm, and its absorbance was recorded as approximately 0.70 (air as the blank). For the analysis, the samples, including the placebo, the curcumin-loaded lipid nanocarriers, and an aqueous curcumin dispersion (containing an amount of curcumin equivalent to that in the curcumin-loaded lipid nanocarriers), were maintained at 34°C (to simulate skin surface temperature). At 1-h intervals over 8 h, 250 µL aliquots were collected and subsequently diluted to a final volume of 1 mL with an ethanol: water (90:10 v/v) solution. The resulting mixture was centrifuged at 13,520 rcf for 30 min at room temperature. A 100 μL volume of this solution was added to 1.9 mL of a diluted ABTS^+^ solution, and the mixture was shaken for 7 min. The absorbance was then measured at 734 nm. A solution without a sample worked as a control. Antioxidant capacity was calculated from a Trolox standard curve, and results were expressed as Trolox-equivalent antioxidant capacity (TEAC, μmol/mL Trolox) over time.

### Microbial Inhibition Studies With Multi-resistant Bacteria Applying Photodynamic and Sonodynamic Therapies

#### Microbial Culture

The antimicrobial effect of curcumin, alone and in combination with PT and ST, was demonstrated against two bacterial strains: *Pseudomonas aeruginosa* and Methicillin-Resistant *Staphylococcus aureus* (MRSA). Both clinical strains were analyzed and compared using primary and secondary phenotypic biochemical tests with *S. aureus* ATCC 43300 and *P. aeruginosa* ATCC 27853. The strains were supplied by the *Bacteriological Diagnostics Service* at *Facultad de Estudios Superiores Cuautitlán, UNAM*. To test the antimicrobial effect, strains were cultured on Mueller–Hinton agar and incubated at 37°C for 24 h.

#### Microbial Inhibition Tests

The viable count procedure was carried out in accordance with the Mexican standard NMX-BB-040-SCFI-1999 [22]and the USP [23]. To prepare the inoculum, bacteria were taken from the culture plates mentioned in "Microbial Culture" using an inoculating loop and dispersed in 10 mL of sterile physiological saline solution (0.9% w/v) until the turbidity matched that of the 0.5 McFarland nephelometer (equivalent to an approximate bacterial concentration of 1.5 × 10^8^ CFU/mL). Subsequently, six decimal dilutions were prepared, and 100 µL from the last three dilutions was inoculated onto Standard Methods Agar plates. Finally, the plates were incubated at 37°C for 24 h. Each determination was performed in duplicate on two different days.

From the tubes of both strains matched to the McFarland nephelometer, 0.5 mL were put in contact with an equal volume of: (i) curcumin aqueous dispersion, (ii) curcumin-loaded lipid nanocarriers, or (iii) placebo lipid nanocarriers, in a concentration range of 25–50 µg/mL and for a time interval of 11 to 36 min without any irradiation. Dilutions and inoculation were performed as previously described. To assess the effect of PT on the bacterial suspension, the previously described procedure was followed, applying 475 nm light from a prototype lamp developed by the Department of Engineering (FESC-UNAM), whereas for ST, a commercial ultrasound system (AUKEWEL® ultrasound equipment, model AK-CS-02, 1 MHz) was used. In both cases, the test conditions and equipment characteristics are described in the Supplementary Material (Figures S7 and S8). After the therapy (PT or ST) was administered, a series of decimal dilutions was performed, as previously described. Each determination was carried out in quadruplicate. Finally, calculations of viable counts were performed using Eq. (4):

4 $$\frac{CFU}{ml}=\frac{(Average~CFU )(Dilution~tube)}{0.1~mL}$$

where *Average CFU* was the number of microbial colonies counted on agar plates (between 30 and 300 CFU); *Dilution tube* corresponds to the dilution where the average CFU was counted (e.g., 10^5^ or 10^6^), and 0.1 mL was the volume taken from the dilution tube for plating on agar plates. Once the CFU/mL values were determined, the data were log-transformed to calculate the % microbial inhibition ± SD for each formulation.

### Cytotoxicity Assays

The toxicity of curcumin, placebo lipid nanocarriers, and curcumin-loaded lipid nanocarriers was assessed using the MTT assay on HaCaT keratinocytes. Tests were performed as described below, with or without PT or ST. Briefly, 10^4^ cells seeded 24 h earlier were plated into 96-well plates containing IMDM medium. Curcumin solutions in DMSO and lipid nanocarrier suspensions were added at concentrations of 25, 50, and 75 µg/mL and incubated at 37°C with 5% CO_2_ for 24 h. Once the time elapsed, the systems were removed, and MTT reagent (0.5 mg/mL) was added, after which they were incubated under the same conditions. After incubation, MTT was removed, and 100 µL of DMSO was added. The plate was then shaken at 200 rpm for 15 min. The absorbance was measured at 492 nm using a plate spectrophotometer (Epoch BioTek® Instruments Inc., USA). Identical studies were performed under the best antimicrobial PT conditions (blue light at 20 mW/cm^2^ for 36 min). As before, experiments were performed using the best antimicrobial ST conditions (3.5 W/cm^2^ for 20 min).

Alternatively, for ST, the toxicity of curcumin, placebo lipid nanocarriers, and curcumin-loaded lipid nanocarriers was evaluated using an erythrocyte hemolysis assay [24]. Briefly, 5 mL of blood was collected in anticoagulant tubes and centrifuged at 1700 g for 5 min; the supernatant was then discarded. Subsequently, the cells were washed with 2 mL of PBS pH 7.42 and centrifuged under the same conditions. The washes were repeated until a translucent supernatant was obtained. Then, the erythrocyte suspension (1% (v/v)) was treated with the formulations (placebo lipid nanocarriers, curcumin in DMSO, or the curcumin-loaded lipid nanocarriers), with or without ST (3.5 W/cm^2^ for 20 min). Several controls were evaluated, including i) Triton X10 20% v/v (positive control), ii) PBS pH 7.42 (negative control), ultrasound (ST) only, DMSO, and ST + DMSO. The systems were incubated for 24 h and centrifuged at 1700 g for 10 min. The supernatant was collected and diluted 1:10 in PBS in 48-well plates, and absorbance was measured at 405 nm using a plate spectrophotometer (Epoch BioTek® Instruments Inc., USA). The samples were read by adjusting with the corresponding blanks. All the studies were carried out in sextuplicate.

### Analytical Method for the Quantification of Curcumin

To determine the amount of curcumin in lipid nanocarriers and in the receptor medium during the release and permeation studies, a spectrophotometric method was employed using a Hitachi spectrophotometer (model U-5100, Japan). A calibration curve was constructed in the range of 0.5118–4.662 µg/mL in an ethanol:water mixture 1:1 (v/v), with readings taken at 429 nm. For the permeation studies, to simulate the test conditions, a 1:1 (v/v) ethanol:water solution was used, which was previously placed in contact with the inner surface of the skin for 24 h. The resulting solution was filtered, and the calibration curve was prepared with it. The method was validated by evaluating specificity, linearity, repeatability, and accuracy. Information on method validation is provided in the Supplementary Material (Table S1 and Figure S1).

### Statistical Analysis

The results were expressed as the mean ± standard deviation (SD). Statistical analysis was carried out using Student’s t-test or one-way ANOVA, followed by a post-hoc Tukey test. The significance level was set at *p* < 0.05.

## Results and Discussion

### Preparation and Characterization of Curcumin-loaded Lipid Nanocarriers

Although the best formulation was selected according to an experimental design (see Tables S2-S4 and Figures S1-S4, Supplementary material), during lipid nanocarrier preparation, other factors were also relevant when deciding which formulation would be used for the rest of the tests: i) The phospholipid source was the main factor that affects both particle size and PI. When standard soy lecithin (SSL) was used, the particle size was three times larger compared to formulations containing water-dispersible lecithin (WSL) (Table S2, Supplementary Material); furthermore, SSL has a high tendency to become contaminated with fungi; ii) there was no difference between using ethanol or isopropanol; iii) formulations containing 0.5 mg/mL of curcumin were more unstable and precipitated on the third day; iv) although formulations with PP123 presented better technological characteristics (smaller size and PI), fungal contamination was observed and film hydration was more complicated compared to PF127. Thus, a formulation containing curcumin (CU) and WSL was chosen and coded as CUWSL. In this formulation, PF127 was used as a stabilizer and edge activator, and ethanol as the solvent (formulation XIII, Table S2 of the Supplementary Material).

**Fig. 1:**
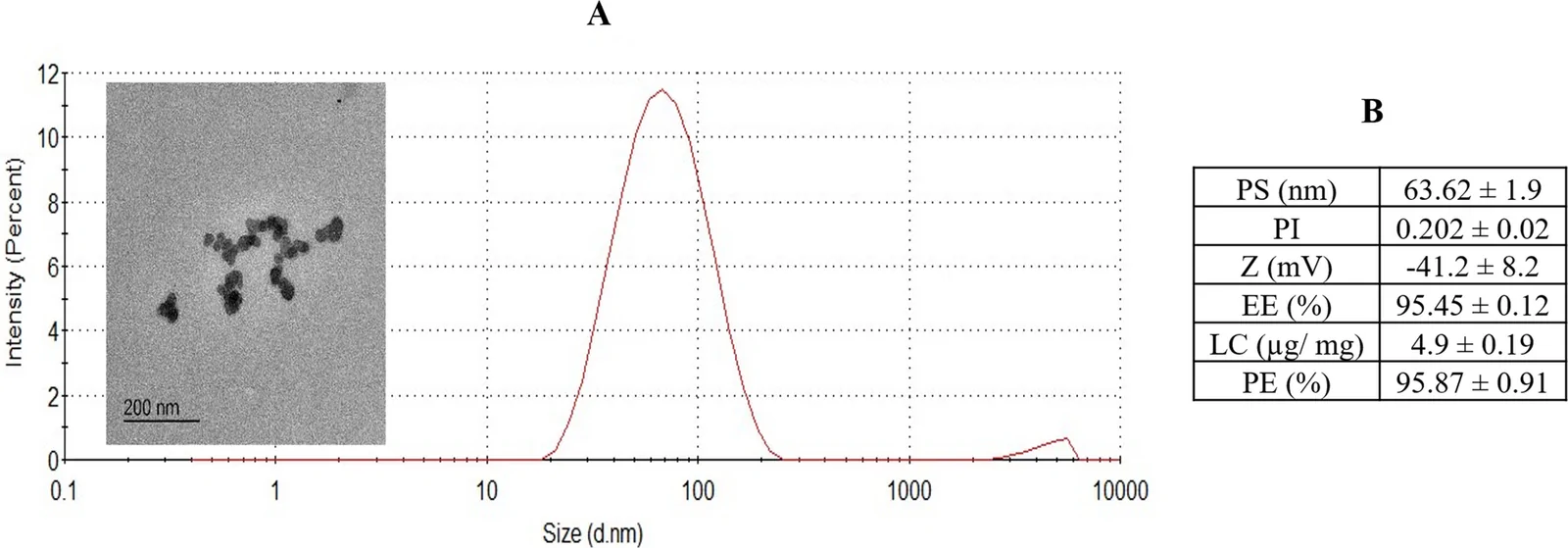
Characteristics of the CUWSL formulation (curcumin-loaded lipid nanocarriers with water-dispersible lecithin and PF127). (A) TEM micrography and size distribution. (B) Physicochemical properties: Particle size (PS), Polydispersity Index (PI), Z potential (Z), Entrapment Efficiency (EE), Loading Capacity (LC), Process Efficiency (PE). Results are shown as the average ± standard deviation (n = 3).

In this work, quasi-spherical particles were observed, with an average size of 63 nm and a polydispersity index (PDI) of 0.202, indicating a uniform particle population (Fig. 1). As various reports confirm, the composition of the particles determines their characteristics [25–28]. The small size can produce a high curvature, creating stress that slightly distorts the shape. Nevertheless, given the results of this study, it is not possible to determine the curvature-induced effects arising from the combination of these factors [29]. In fact, none of the formulations prepared with WSL exceeded 80 nm, even at higher curcumin concentrations (see Table S2, Supplementary material), which may be due to WSL composition and its interactions with curcumin. According to US4174296A-WLS Composition [30], surfactants such as tertiary amine oxides, alkyl alcohol sulfate salts, alkylamidopropyl betaines, sodium alkenesulfonates, alkyl sulfoacetates, and sulfosuccinate monoalkyl amide disodium salt could be incorporated. Therefore, these surfactants could reduce interfacial tension and thereby decrease particle size. On the other hand, it could also favor the incorporation of curcumin into lipid nanocarriers, which explains the high entrapment efficiency (EE) (> 95%; Fig. 1B), making it a better candidate for skin administration [31, 32]. For lipophilic molecules, entrapment efficiency also depends on the concentrations of phospholipid and stabilizer [33]. By contrast, all SSL-based formulations had sizes greater than 130 nm (Table S2, Supplementary Material) and significantly lower EE (ranging from 52 to 95%, data not shown), depending on the curcumin concentration. As shown in Fig. 1B, a Z-potential of −41 indicates that the CUWSL formulation exhibits good physical stability [34].

**Fig. 2:**
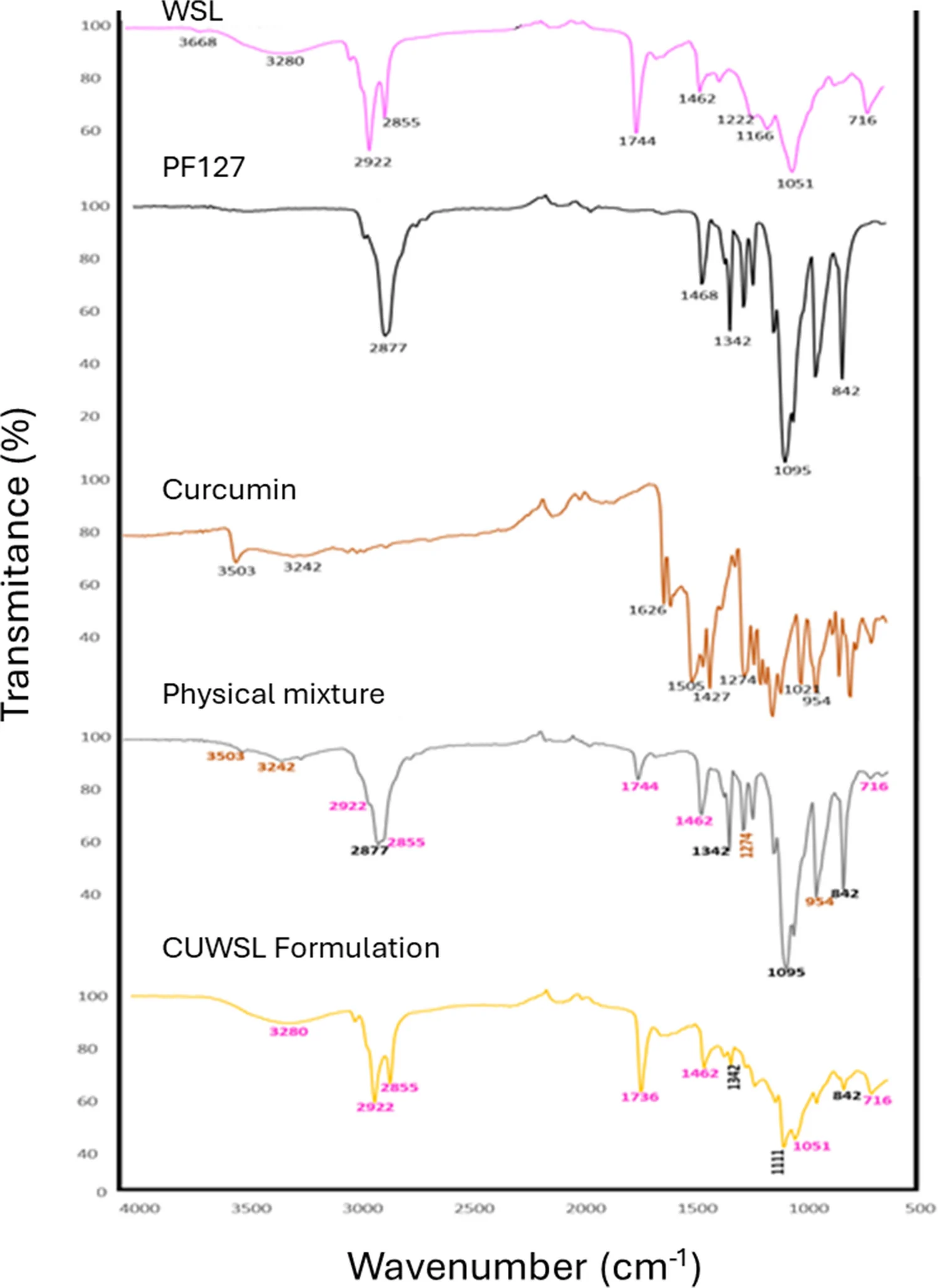
Infrared spectra for the CUWSL formulation, the physical mixture, and the raw. WSL = water-dispersible lecithin, PF127 = Pluronic® F-127.

Figure 2 presents the infrared spectrum of the raw materials, a physical mixture, and the CUWSL formulation. Regarding the physical mixture, the signals correspond to the three components of the mix: WSL (2922, 2855, 1744, 1462, and 716 cm^−1^), PF127 (2877, 1342,1095, and 842 cm^−1^), and curcumin (3503, 3242, 1274, and 954 cm^−1^) [35, 36]. The spectrum of the CUWSL formulation showed fewer bands than that of the physical mixture, with some signals attributed to curcumin and PF127 disappearing or being displaced. The interaction between curcumin and the formulation components may explain this.

**Fig. 3:**
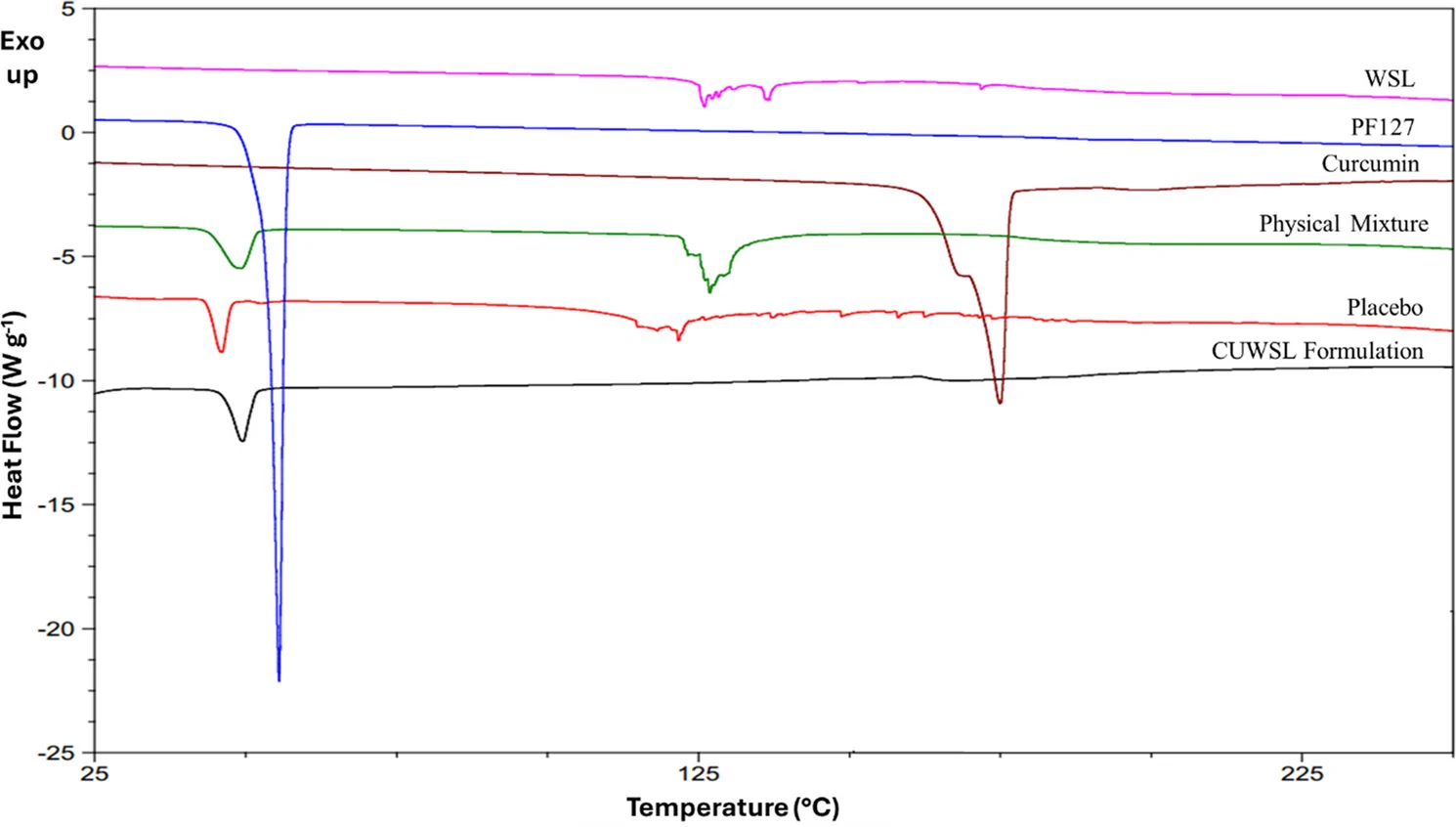
Thermograms for the CUWSL formulation, the physical mixture, the placebo, and the raw materials. WSL = water-dispersible lecithin, PF127 = Pluronic® F-127.

The DSC thermograms of the raw materials, the physical mixture, the CUWSL placebo, and the CUWSL formulation are shown in Fig. 3. WSL exhibited a small signal attributable to the Tm at 40°C. In contrast, from 116 to 171°C, various endothermic signals were observed, attributed to phospholipids and to components that render this lecithin water-dispersible. PF127 presents a high endothermic signal at 55.95°C, corresponding to the melting point [36]. Curcumin exhibited endothermic signals between 165 and 175°C corresponding to its melting point, as well as the melting point of any curcuminoids that may be present [37]. The physical mixture showed endothermic signals from 110 to 140°C, which are the product of the overlap of WSL signals with curcumin, so an isolated experiment with the physical mixture adding a larger amount of curcumin was performed, finding that the signal ranging from 110°C to 140°C increased (data not shown). Thus, it was deduced that the curcumin signal shifted and overlapped in this temperature range. Analysis of the placebo thermogram revealed that the signals associated with PF127 and WSL shifted upon particle formation. In the case of the CUWSL formulation, only the signal attributed to PF127 was visible (shifted, as in the physical mixture and placebo, to a lower temperature). In both physical mixture and CUWSL formulation, the curcumin signal was not evident, which may be due to the low curcumin content or, as mentioned, to the fact that it may be molecularly dispersed [38].

The intermolecular interactions of curcumin with WSL and PF127—as evidenced by both FTIR and DSC analyses—affect the nanocarrier matrix density, which is directly related to the TEM observations (Fig. 1). Typically, in negative staining with phosphotungstic acid, organic nanovesicles (such as liposomes) appear as bright structures against a dark background. In our case, the CUWSL formulation appears as a completely dark, electron-dense entity. A similar contrast, using phosphotungstic acid, was recently reported by Kodous *et al*. [39], who found that encapsulating β-carotene (a lipophilic compound) in lecithin-based bilayers significantly alters the membrane surface packing properties due to the presence of β-carotene in the hydrophobic region of the vesicular bilayer, increasing the electronic contrast and resulting in black-stained vesicles. In our case, the inclusion of curcumin within the CUWSL formulation can disrupt the typical lipid arrangement, thereby allowing the stain to penetrate or bind to the nanostructures, rendering them dark against a lighter background. Authors such as Hardiansyah *et al*. [40] also observed dark structures on a light background in TEM images of curcumin-loaded PEGylated liposomes after staining with phosphotungstic acid.

### *In Vitro* Release Studies

**Fig. 4:**
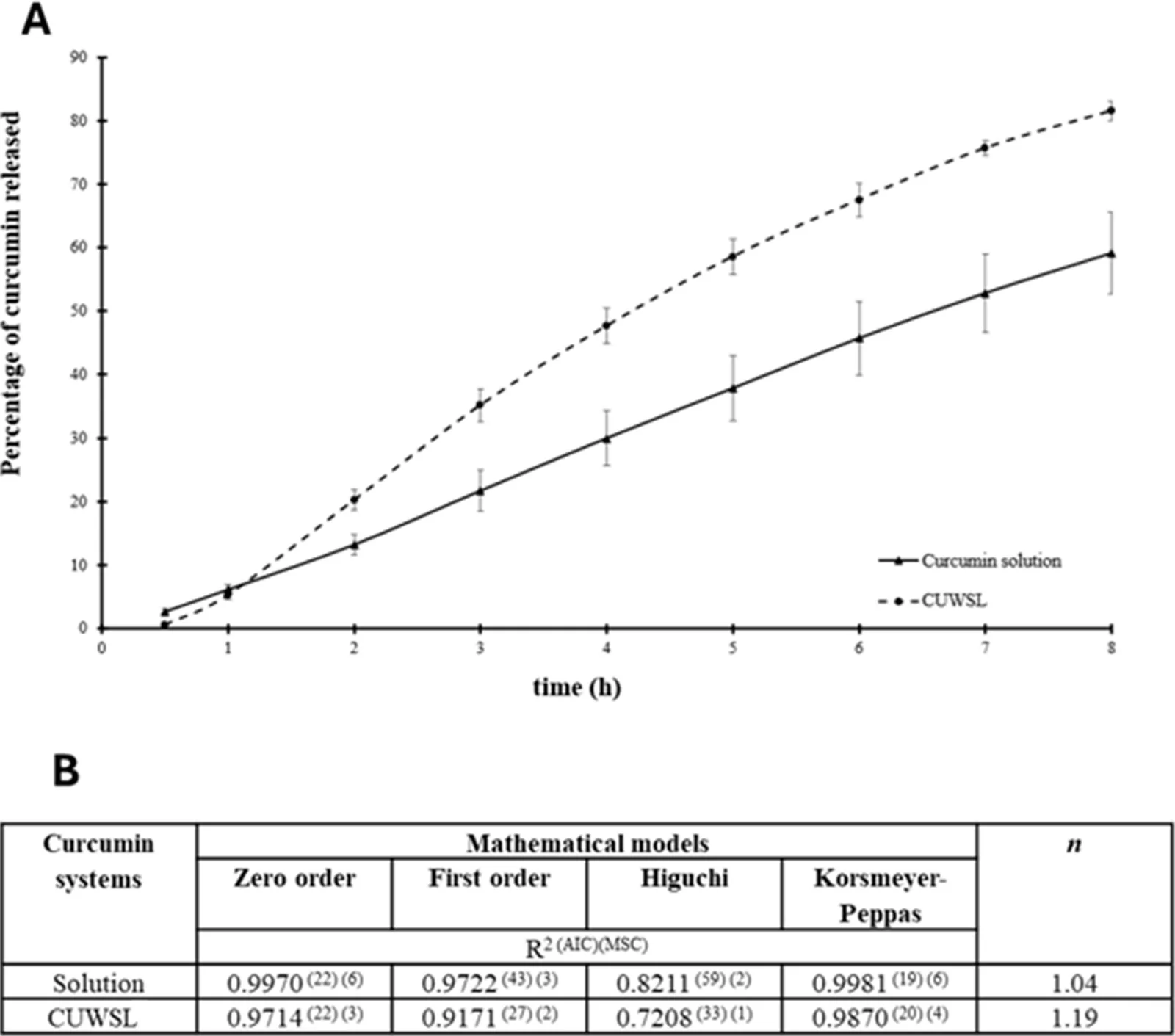
**(A)** Release profile of curcumin from the CUWSL formulation, and for comparative purposes from a solution of curcumin in ethanol:water 1:1 (v/v). Both the formulation and the solution contain the same amount of curcumin. Error bars represent standard deviation (n = 3). **(B)** Mathematical models used to define the kinetics and/or the release mechanisms. R^2^ = Determination coefficient, AIC = Akaike Criteria, MSC = Selection criteria. “*n*” represents the release exponent for the Korsmeyer-Peppas model.

Figure 4A shows the release profiles of curcumin from the CUWSL formulation, and for comparative purposes from a solution of curcumin in ethanol:water 1:1 (v/v). As shown, a greater amount of curcumin was released from the CUWSL formulation (81.5%) into the receptor medium compared to the solution (59.1%). This is attributable to the fact that the WSL components, in conjunction with PF127, as shown in Figure S10 (Supplementary Material), enhance curcumin solubility in the medium. This may favor curcumin migration from the lipid nanocarriers to the water–ethanol interface, thus explaining the increased rate of release. On the other hand, because the release medium is a hydroalcoholic solution, it can compromise the integrity of lipid nanocarriers, breaking them and promoting curcumin release. As shown in Fig. 4B, the zero-order and Korsmeyer-Peppas models best described curcumin release into the receptor medium, with the highest R^2^, the lowest AIC, and the highest MSC. Since the value of “*n*” for this model is close to 1, this confirms the zero-order release [41].

### *In Vitro* Permeation Study and Distribution in the Skin

Curcumin transport through the skin was assessed using *in vitro* permeation tests on both intact and wounded skin. These tests were performed using the CUWSL formulation and, for comparison, a hydroalcoholic curcumin solution. It is worth noting that during the permeation studies, no curcumin was detected in the receptor medium. Therefore, it was assumed that under the test conditions, neither free curcumin nor curcumin included in the CUWSL formulation was able to cross the skin and reach the receptor solution. Therefore, permeation profiles are not shown in this manuscript. The results align with those reported by Pamunuwa *et al*. [42], who found that curcumin included in liposomes was deposited on pig ear skin without permeating into the receptor medium.

**Fig. 5:**
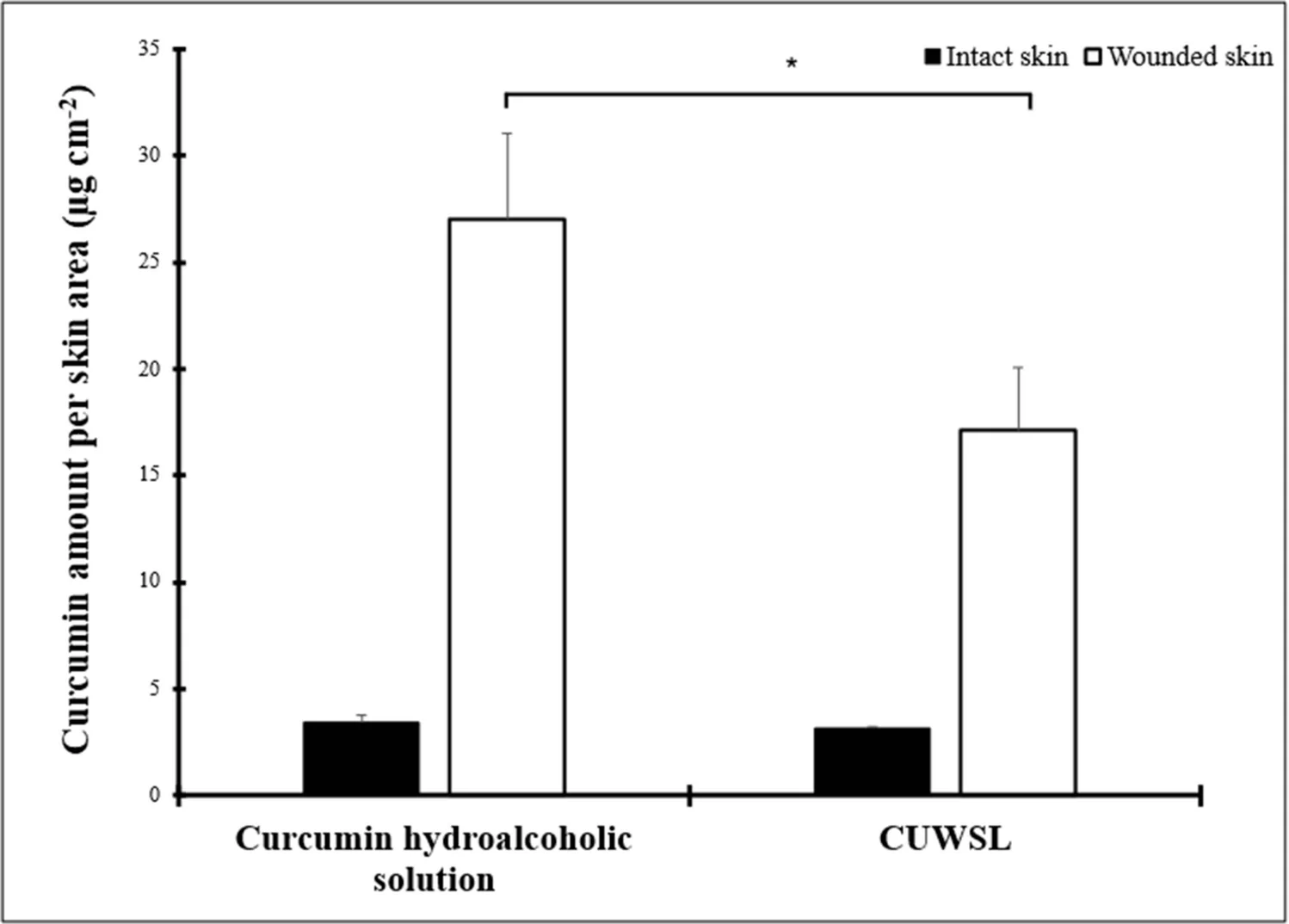
Amount of curcumin retained in intact and wounded skin at the end of the permeation studies (*p < 0.05). Values are indicated as mean ± SD (*n* = 3).

**Fig. 6:**
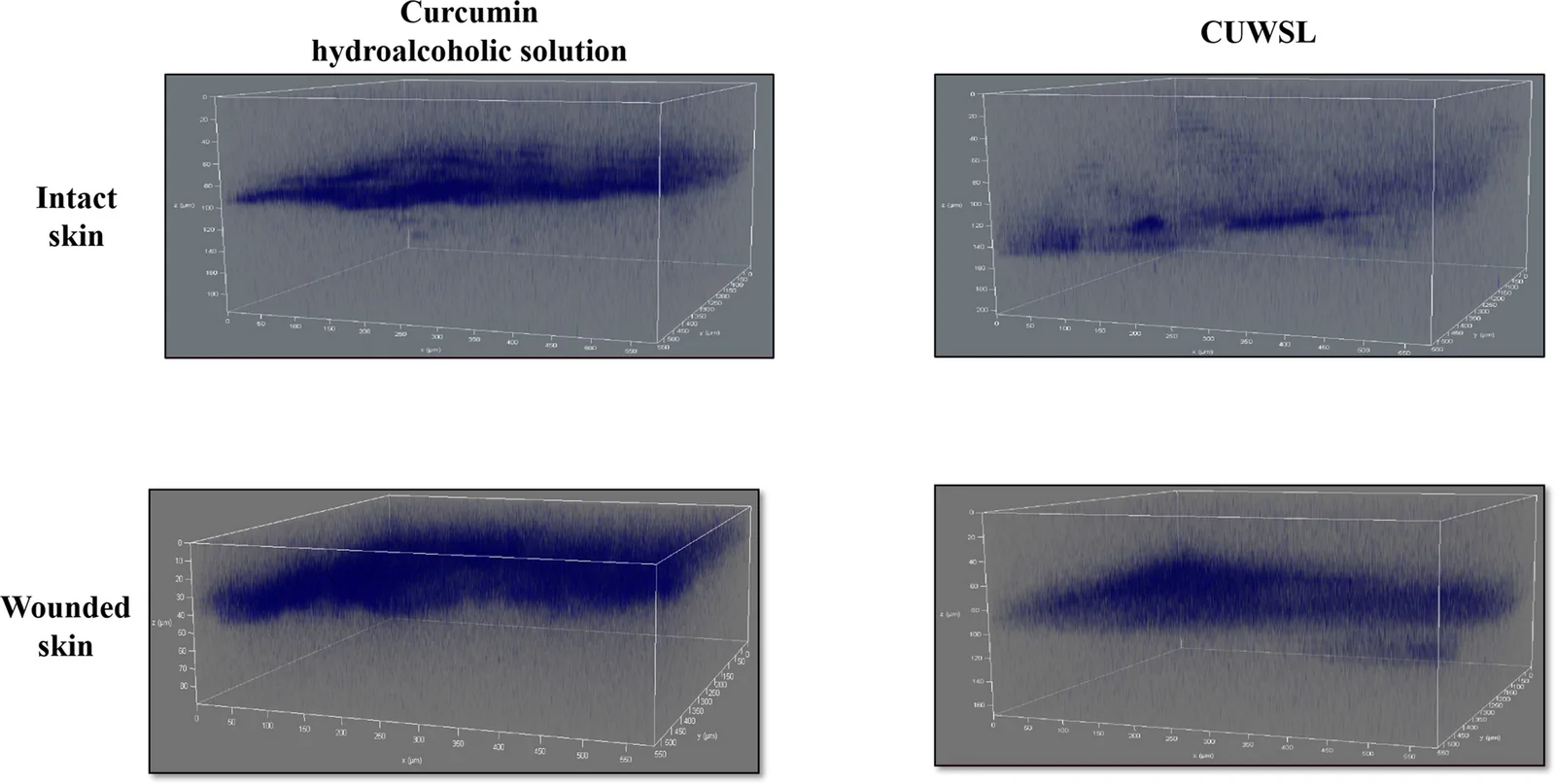
3D images obtained by confocal laser microscopy, showing the distribution of curcumin in hydroalcoholic solution and in the CUWSL formulation throughout intact and wounded skin.

At the end of the permeation test, the skin was recovered with the aim of determining: (i) the amount of curcumin retained in the tissue, proceeding to its extraction; (ii) the distribution of curcumin in the skin using confocal laser microscopy. The results showed higher curcumin deposition in the wounded skin (lacking the stratum corneum) with the hydroalcoholic solution than with the CUWSL formulation (Fig. 5). No significant difference (*p* > 0.05) was observed in the amount of curcumin retained in intact skin from either the solution or the CUWSL formulation. The images obtained by confocal laser microscopy (see Fig. 6) showed that the CUWSL formulation reached deeper regions compared to the solution, both for intact skin (CUWSL formulation 90–140 µm > curcumin solution 40–100 µm) and for the wounded skin (CUWSL formulation 40–100 µm > curcumin solution 0–50 µm). Thus, considering all the results, both in terms of the amount retained and the penetration distance, it can be said that, as expected, the stratum corneum limited the amount permeated, so when it was removed (wounded skin), the amount permeated increased for both the solution and the CUWSL formulation, being greater for the solution. Nevertheless, the CUWSL formulation demonstrated a greater capacity to reach deeper regions compared to the solution.

Lipid nanocarriers and ethanol can act through different mechanisms to promote curcumin penetration: On the one hand, the composition of lipid nanocarriers, i.e., lecithin and PF127, can act as penetration enhancers; on the other hand, the well-known promoting effect of ethanol. We also cannot ignore that PF127, while acting as a stabilizer, can serve as an edge activator, potentially providing some flexibility to the lipid nanocarriers and also promoting particle penetration. Lipid nanocarriers were negatively charged (see Fig. 1B) and were able to penetrate the skin. This finding aligns with reports from other authors, who have found that anionic nanoparticles can penetrate the skin. This occurs because the negative charge on nanoparticles repels negatively charged skin lipids, facilitating their entry into the deeper layers of the skin [43].

Because the primary challenge in administering curcumin through the skin is achieving adequate penetration, many studies, including ours, have focused on formulating curcumin in various carriers, particularly nanosized ones. In this sense, most reports agree that nanocarriers enhance curcumin penetration into the skin [44–49] Other authors, such as Campani *et al*. [50], reported that free curcumin was preferentially retained in the dermis rather than when it was included in liposomes, whereas in the epidermis, the retention levels were similar for both.

### *In vitro* Occlusion Factor and Transepidermal Water Loss (TEWL)

As shown in Table I, after 1 h of contact with intact skin, the CUWSL formulation increased TEWL by 49.7%. This implies that the system is not only non-occlusive but also promotes water transport to the skin surface. This can be explained by the interaction of the components of the CUWSL formulation with the intercellular lipids of the skin, which can act as penetration enhancers, as already mentioned in Sect. "*In Vitro* Permeation Study and Distribution in the Skin" and as explained by Gómez García *et al*. [46], who reported that some components of nanohyaluronan-glycerosomes can alter the intercellular lipids of the skin, promoting the passage of other vesicles. The effect of these enhancers is reciprocal: they promote the entry of drugs and/or carriers and allow greater water to escape from the interior to the skin surface [51]. Regarding wounded skin, the initial TEWL value was higher than that of intact skin (71.88 g h^−1^ m^−2^) due to the absence of the stratum corneum. Following contact with the CUWSL formulation, TEWL increased by only 7.4% (77.18 g h^-1^m^-2^). This modest increase is attributed to the absence of a permeability barrier and, as noted above, to the formulation’s lack of occlusive effect. These results coincide with those obtained when measuring the occlusion factor (F), which was less than 5% (Table I), indicating that the occlusion effect was minimal compared to particulate nanocarriers such as Solid Lipid Nanoparticles or Nanostructured Lipid Carriers, for which values between 30 and 40% were reached according to the studies performed by López-García and Ganem-Rondero [17].

**Table I:** Results of the *In Vitro* Transepidermal Water Loss (TEWL) and Occlusion Tests. (A) TEWL for Intact and Damaged Skin in Contact with the CUWSL Formulation. The Ratio of the TEWL Value After 1 h of Treatment to the Initial TEWL is Reported. (B) *In Vitro* Occlusion Factor (F) Using Whatman® No. 42 Paper. The Results are the Average of Three Determinations ± Standard Deviation

(A)TEWL		
**Intact pig ear skin**		
Initial TEWL (g h^−1^ m^−2^)	1 h TEWL (g h^−1^ m^−2^)	1 h TEWL/Initial TEWL
21.07 ± 1.82	31.56 ± 0.97	1.519 ± 0.279
**Wounded pig ear skin**		
71.88 ± 2.03	77.18 ± 1.08	1.07 5 ± 0.038
***(B)In vitro*** **occlusion factor (F) expressed as a percentage**		
8 h	24 h	48 h
4.69 ± 1.59	4.68 ± 0.63	4.56 ± 1.05

### Stability Studies

**Fig. 7:**
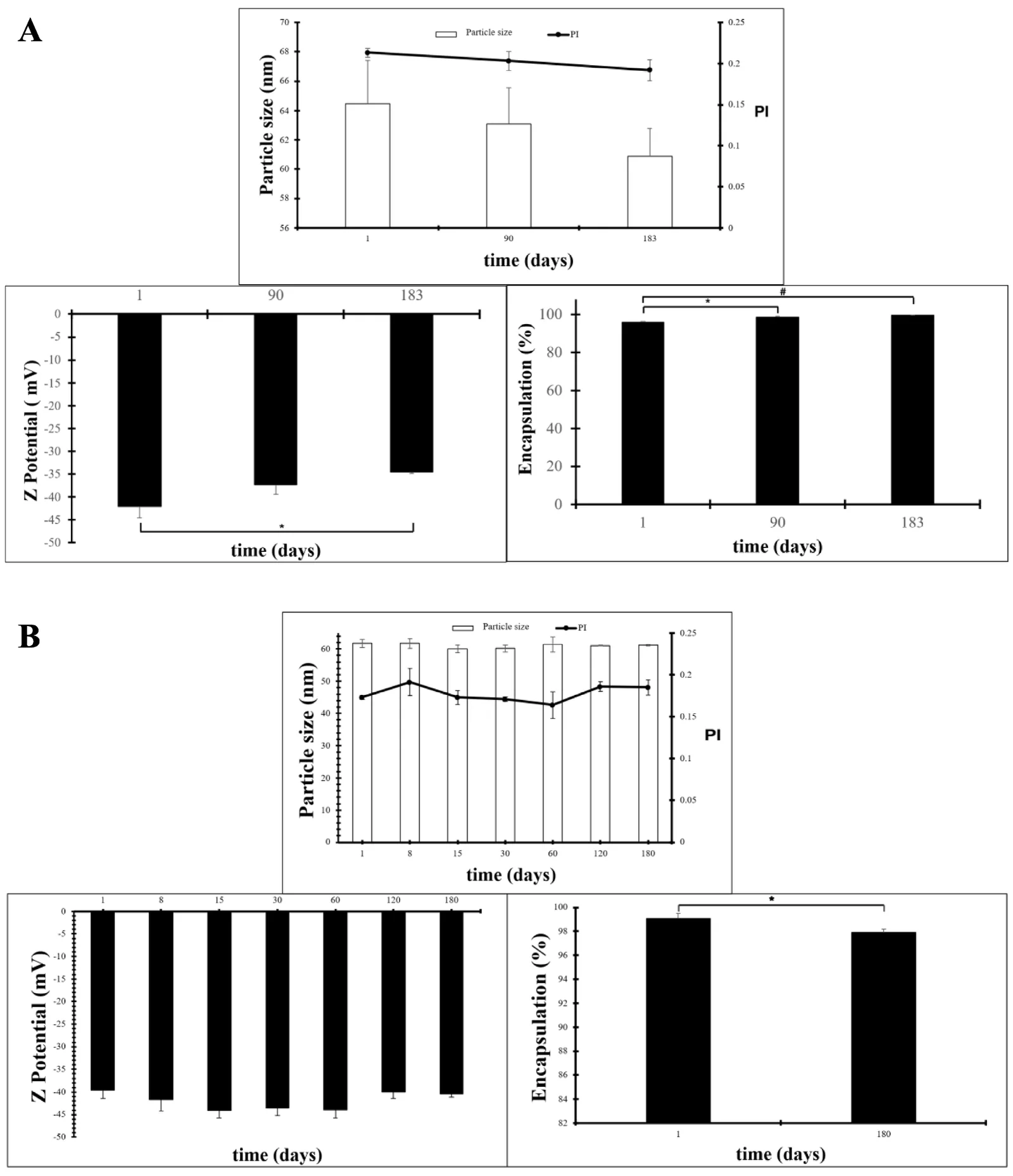
Results of the stability tests for the CUWSL formulation. (A) Long term (5°C, 12 months^1^). (B) Accelerated (25°C, 60% RH, 3 and 6 months). Values are indicated as mean ± SD for *n* = 3 (*p < 0.05). ^1^Only results up to 6 months are shown. After this time, fungal contamination was observed.

Figure 7 presents the results of the accelerated and long-term stability tests for the CUWSL formulation. Across the two conditions, all measured parameters remained essentially constant over 6 months; for example, size ranged from 60 to 64 nm, PI from 0.191 to 0.213, Z-potential from −34 to −44, and encapsulation from 96 to 99.5%. Although statistically significant differences were found for some parameters, these differences are attributable to the very low variability among the measurements. Overall, the formulation remained stable; however, fungal contamination was observed after 6 months. Eleraky *et al*. [52] conducted a long-term stability study over 60 days, demonstrating that vesicle size, PI, and Z potential remained unchanged throughout this period. According to what has been reported in the literature, the predominant tautomeric form of curcumin in most nonpolar organic solvents is the enol form. In this case, curcumin is embedded within the lipid nanocarrier matrix, thereby maintaining its enol form. Therefore, this form is assumed to be maintained when analyzing the formulations over the long term. This tautomer was responsible for the pro-oxidant activity because it could generate ROS [53, 54]. This is discussed in more detail in the *Photodynamic and Sonodynamic Therapies* Section.

**Fig. 8:**
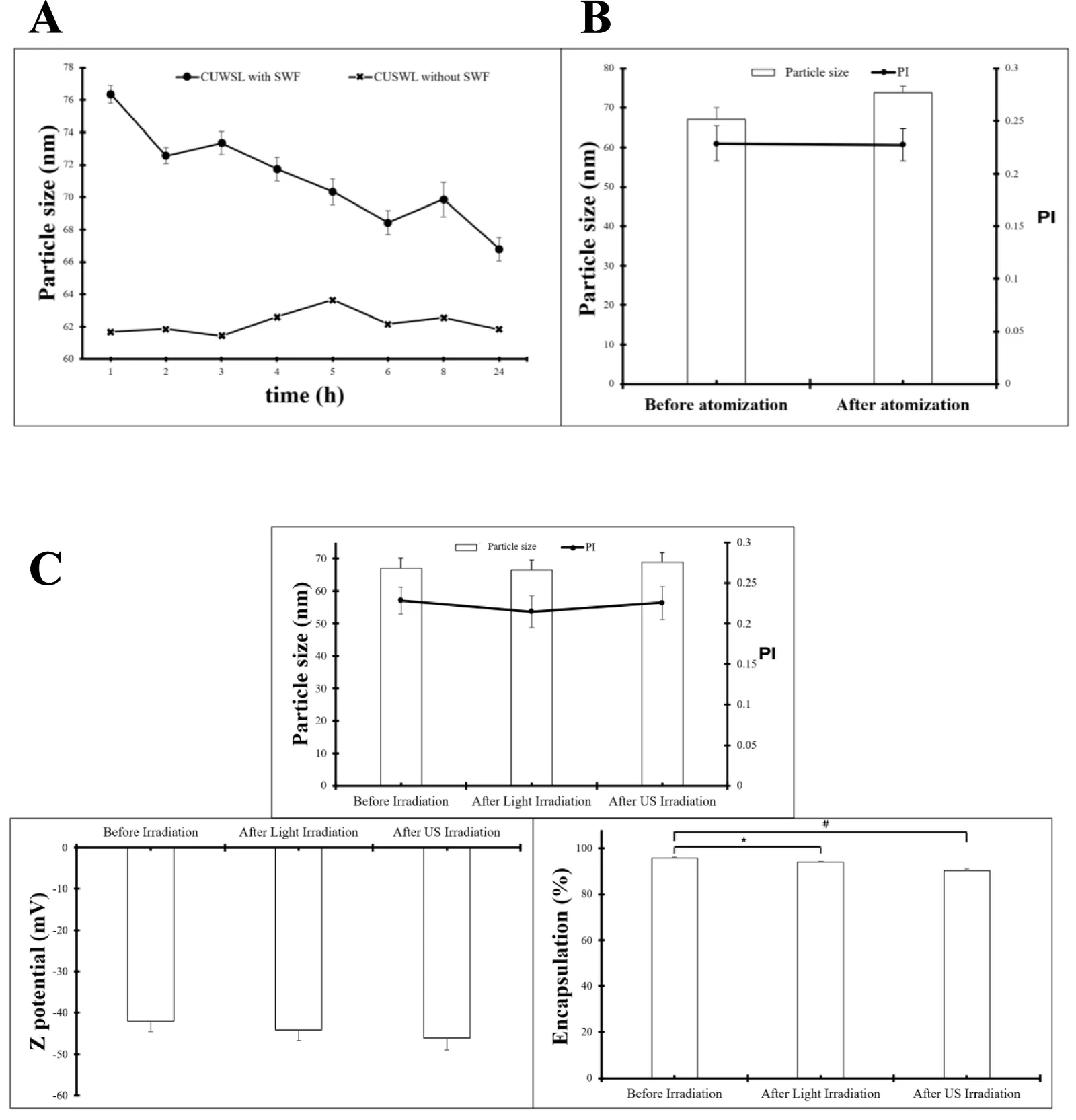
Results of the stability test for the CUWSL formulation. (A) In contact with simulated wound fluid (SWF), and for comparative purposes, without SWF, maintaining the CUWSL formulation at 34°C. (B) after atomization. (C) Irradiating with light and ultrasound (US). Values are indicated as mean ± SD for *n* = 3 (* and # *p* < 0.05).

Figure 8A shows the results of lipid nanocarriers’ stability against simulated wound fluid (SWF). As observed, upon contact with the SWF, particle size increased by approximately 12 nm (average size of 76 nm) and then decreased over the 24-h study period to approximately 67 nm. It has been reported that nanocarriers tend to adsorb proteins and other biomolecules upon contact with biological fluids, thereby increasing the surface energy. These proteins, collectively known as the protein corona, modify the surface features of the nanocarriers, resulting in biological behaviors different from those they would exhibit in their original state, thereby affecting their trajectories within biological systems [55, 56]. Generally, it is thought to shield cells from the reactive surfaces of nanocarriers and enhance safety in nanomedicines [57, 58]. Upon contact with blood, proteins immediately interact with nanocarriers via noncovalent interactions, including hydrogen bonds, van der Waals forces, and electrostatic attractions, thereby forming a protein corona. This explains the increase in their size upon contact with the SWF. However, over time, lipid nanocarriers decreased in size, which can be attributed to the different ways in which the protein corona interacts with them: (i) the formation of the protein corona can affect the integrity of the lipid nanocarriers; (ii) protein adsorption alters the osmotic balance between the interior of the lipid nanocarriers and the external fluid. When the protein corona forms, the osmotic pressure inside lipid nanocarriers increases, causing water to leave them and thereby reducing their size [59]. In addition, blood lipids maintain an exchange balance with the lipids of the vesicular matrix. The stability of negatively charged liposomes varies with their composition; those composed of a single phospholipid type are unstable due to their high affinity for proteins. In contrast, those that include phosphatidylcholine and other phospholipids are more stable since they adhere less to proteins [60]. Furthermore, as shown in Fig. 8A, lipid nanocarriers without SWF remained stable at 34°C, exhibiting no significant differences over time for at least 24 h.

The process of breaking down large volumes of liquids into droplets is known as atomization. Generally, the atomization process depends on the surface tension, viscosity, and density of the fluid being atomized. If these three factors are high, the resulting droplet size will be large [61, 62]. However, if the droplet size is too small, the lipid nanocarriers risk merging and failing to penetrate the skin. In this work, no significant change was observed in particle size or PI, as shown in Fig. 8B. This may be due to the presence of PF127, which can act as an edge activator, conferring some elasticity to the lipid nanocarriers. In this way, they were able to pass through the node without losing their original size despite the shear. Finally, as shown in Fig. 8C, there were no significant changes in the Z potential, size, or PI of liposomes upon irradiation with light or ultrasound (US). Regarding curcumin content, it decreased by approximately 1.74% with light exposure and by 5.48% with US, with significant differences in both cases compared with untreated lipid nanocarriers. In the case of light, both phospholipids and curcumin can be degraded upon blue-light irradiation (the latter via ROS generation) [63, 64]. US waves are also able to modify the structure of the vesicular matrix, causing the release of curcumin, which in turn can undergo autoxidation reactions in neutral or basic media; in addition, it has been reported that the efficiency of ROS generation by curcumin with US is greater than with blue light [64]. All of this could explain the difference in curcumin content in lipid nanocarriers treated with US and irradiated with blue light.

### Antioxidant Activity

**Fig. 9:**
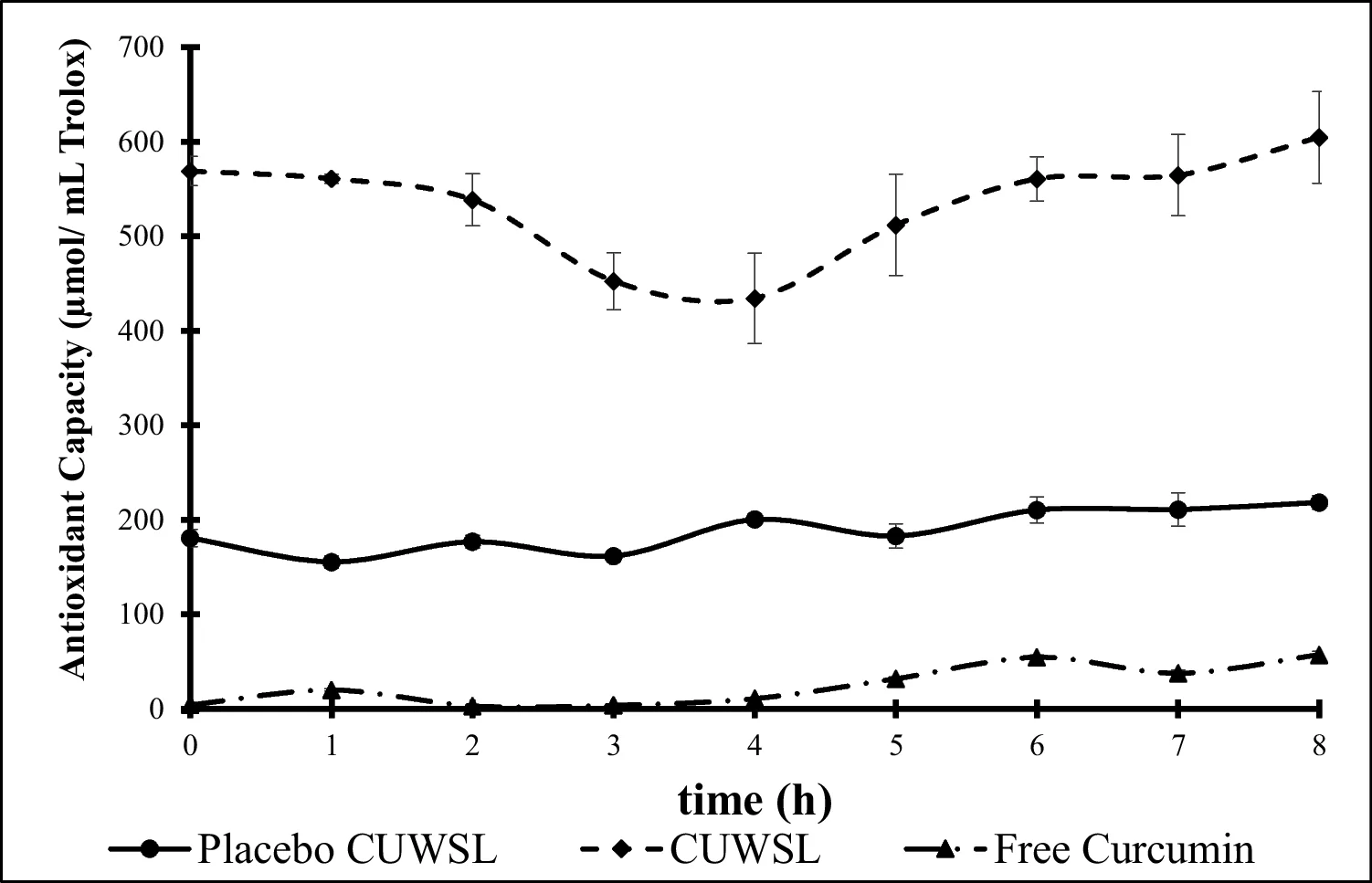
Antioxidant activity of curcumin expressed as Trolox equivalent antioxidant capacity (TEAC) (μmol/mL Trolox) for CUWSL formulation, CUWSL placebo, and free curcumin (curcumin aqueous dispersion). Error bars represent standard deviation (n = 3).

Figure 9 shows the results of the antioxidant capacity for free curcumin, the placebo lipid nanocarriers, and the CUWSL formulation. As shown, curcumin in aqueous dispersion exhibited practically no antioxidant activity until 4 h, after which an increase was observed (p > 0.05), which can be attributed to the degradation products of curcuminoids (e.g., vanillin, ferulic acid, ferulic aldehyde, feruloyl methane) able to displayed antioxidant activity [64–68]. The CUWSL formulation exhibited the highest antioxidant capacity, maintaining it for at least 8 h, whereas the placebo showed only one-third of the CUWSL formulation’s antioxidant activity. In this sense, specific components of WSL may have contributed to the formulation’s antioxidant activity, as some exhibit intrinsic antioxidant and antimicrobial properties, including tertiary amine oxides and N-alkyl betaines [69]. Furthermore, the inclusion of hydrophobic drugs in nanocarriers, facilitates better dispersion in water, thereby improving contact between the drug and free radicals [70].

### Photodynamic and Sonodynamic Therapies

Due to the crisis caused by increasing antimicrobial resistance, alternatives to antibiotic use are being sought. Interest in curcumin as a photosensitizer and sonosensitizer has grown in recent years, owing to its natural origin and multiple benefits in treating various diseases. Its inclusion in lipid nanocarriers is due to the need for a carrier that addresses its low solubility and instability under various external factors. Although photodynamic therapy has been widely used to treat infections, limited light penetration may restrict its application to superficial infections, whereas ultrasound waves can reach deep-seated infections that light cannot. Therefore, one of the main objectives of this work was to evaluate and compare the antimicrobial activity of curcumin, incorporated into lipid-based nanocarriers, in combination with photodynamic therapy (PT) and sonodynamic therapy (ST). Based on antibiogram-like assays, curcumin concentrations of 25–50 µg/mL were determined to be suitable for PT and ST. These concentrations were chosen because, in the absence of treatment (light or US), no significant inhibition was observed against the clinical strains MRSA and *P. aeruginosa*.

**Fig. 10:**
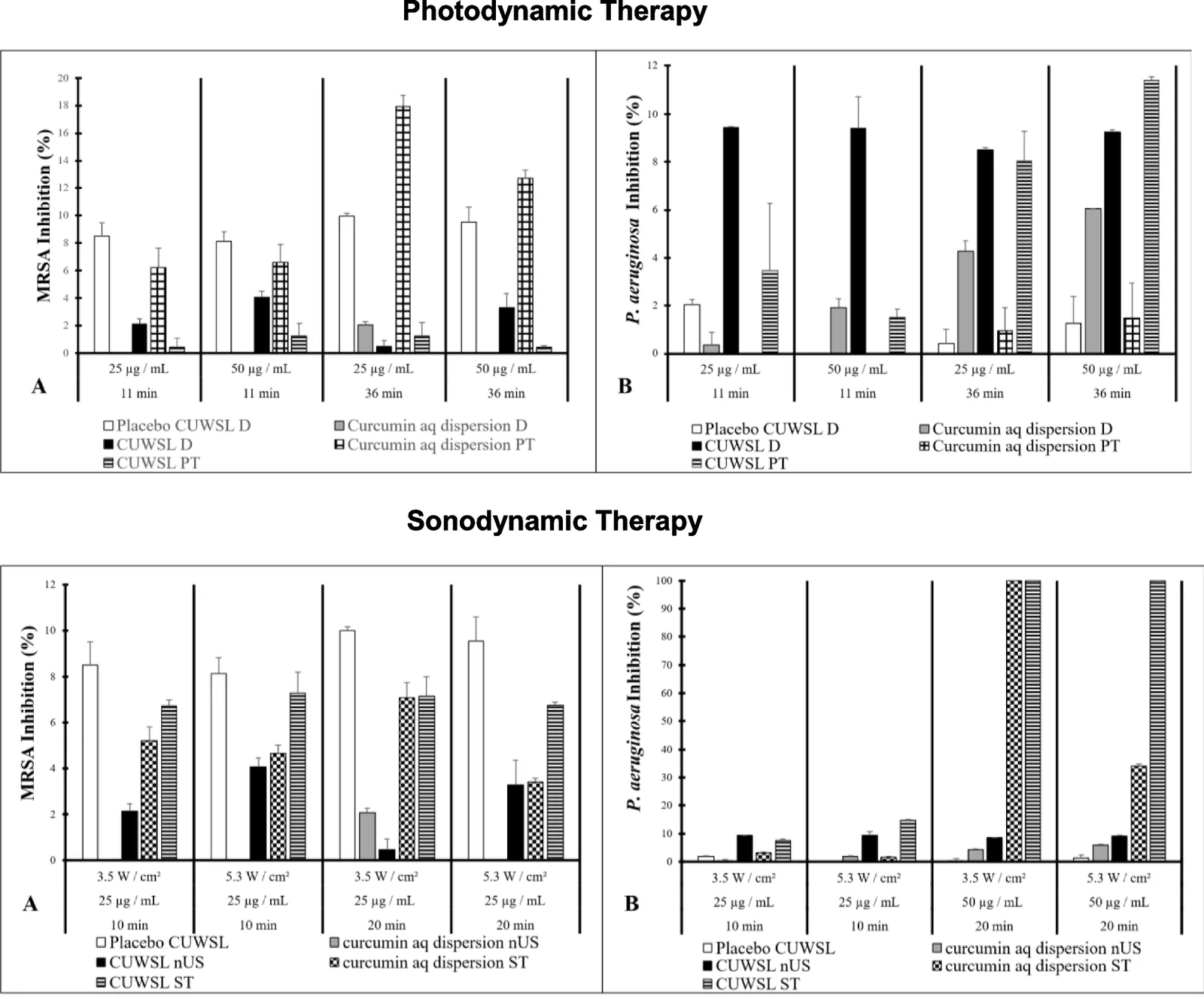
Results of the microbial inhibition rate of curcumin for two multiresistant bacteria using photodynamic therapy and sonodynamic therapy. (A) MRSA. (B) *P. aeruginosa*. PT = Photodynamic Therapy; ST = Sonodynamic Therapy; aq dispersion = aqueous dispersion; D = in Darkness; nUS = without ultrasound. Values are indicated as mean ± SD for *n* = 4.

Looking at the results shown in Fig. 10 for PT (blue light, 475 nm), the following can be commented on: (i) In general, the maximum inhibition was obtained for MRSA with curcumin in aqueous dispersion (18%), and for *P. aeruginosa,* maximum inhibition was observed with the CUWSL formulation (11.4%). In both cases, inhibition increased with increasing curcumin concentration and contact time. (ii) The CUWSL formulation showed inhibitions of less than 2% for MRSA and 11.4% for *P. aeruginosa.* (iii) For *P. aeruginosa*, the CUWSL formulation in darkness (D) inhibited between 8.5 and 9.4%, regardless of the concentration and contact time. (iv) For both MRSA and *P. aeruginosa*, placebos had an inhibitory effect (greater for MRSA, between 8.14 and 10%). (v) Treating the strains with blue light alone (without CUR) had no effect on their viability (data not shown).

Reviewing the ultrasound (US) results, Fig. 10 shows the following for MRSA: (i) Inhibition was very low, being surprising the fact that the highest inhibition (8–10%) was obtained with CUWSL placebo. (ii) The application of US allowed a maximum of 7.28% with the curcumin dispersion and with the CUWSL formulation, with no trend regarding concentration or contact time. (iii) Without applying US (nUS), the maximum inhibition was 4%. Regarding *P. aeruginosa*: (i) In all cases, without applying US (nUS), the inhibition was less than 10%. (ii) Total inhibition (100%) was achieved when applying US both with the curcumin dispersion and with the CUWSL formulation (3.5 W/cm^2^, 50 µg/mL, 20 min), and for the CUWSL formulation (5.3 W/cm^2^, 50 µg/mL, 20 min). Although the microbial inhibition achieved with the CUWSL placebo against MRSA did not exceed 10%, this may be attributed to disruption of the bacterial envelope by the phospholipid bilayer. Exogenous lipids or surfactants can integrate into the *S. aureus* membrane, inducing mechanical stress or localized pore formation. Given that inhibition with the placebo was greater than with the curcumin-loaded formulation, we hypothesize that encapsulating curcumin may alter the surface properties of the lipid nanocarriers, for example, by reducing membrane fluidity [71], thereby mitigating the inherent antimicrobial activity of the lipid vehicle itself.

The complete results of the experimental design used to determine the optimal conditions for ST are provided in the Supplementary Material. It can be concluded that ST enables complete inhibition of *P. aeruginosa*, which is significant given the difficulty of eradicating this bacterium. In contrast, neither PT nor ST favors a high rate of MRSA inhibition. It is important to mention that a treatment with ultrasound alone (without formulation) was performed, applying 3.5 and 5.3 W/cm^2^ for 10 and 20 min, obtaining inhibitions of 15 to 24% for MRSA and 7–9% for *P. aeruginosa*.

In this study, low-frequency ultrasound was employed, whose primary mechanism of action is the formation and violent collapse of microscopic bubbles [72]. This process generates mechanical shear forces and micro-jets that can disrupt bacterial structures. However, it is essential to consider the membrane architecture of Gram-positive and Gram-negative bacteria. Gram-positive bacteria possess a thicker, highly cross-linked peptidoglycan wall, providing significant robustness against the mechanical stress induced by ultrasonic waves. In contrast, Gram-negative bacteria have a much thinner peptidoglycan layer. Although they possess an outer lipid membrane (absent in Gram-positive species), this lipid layer is easily disrupted by ultrasound. Consequently, the thin peptidoglycan layer provides minimal resistance to cavitation, potentially enhancing curcumin permeability [8, 69].

Gram-positive bacteria are more elastic and, therefore, better withstand ultrasonic pressure changes without wall damage. Conversely, Gram-negative bacteria are mechanically more fragile when exposed to microstreaming. Ultrasound can act as a “key” that unlocks the outer membrane of Gram-negative bacteria by creating pores (sonoporation), thereby facilitating curcumin penetration. The application of ultrasound may trigger several processes: (i) excitation of curcumin leading to the generation of reactive oxygen species (ROS); (ii) promotion of curcumin penetration through the lipid membrane of Gram-negative bacteria (sonoporation); and (iii) disruption of the peptidoglycan layer, which is typically thin in Gram-negative bacteria [72–76].

Notably, as shown in Fig. 10B for sonodynamic therapy, curcumin dispersion exhibited greater efficacy at 3.5 W/cm^2^ than at 5.3 W/cm^2^. In this context, the fact that curcumin dispersion works better at a lower intensity (3.5 W/cm^2^) than at a higher one (5.3 W/cm^2^) can be attributed to the following causes: (i) The acoustic shielding effect (or ‘cushioning effect’). At 3.5 W/cm^2^, efficient cavitation is generated, enabling the waves to propagate through the solvent and activate curcumin molecules. When the intensity is increased to 5.3 W/cm^2^, the cavitation generates many bubbles near the ultrasound source (the transducer). These bubbles can be so dense that they act as an acoustic barrier, scattering sound waves rather than allowing them to pass through, thereby preventing the energy from effectively reaching all curcumin molecules in the dispersion [77]. (ii) Free curcumin is directly exposed to the high-pressure shocks and thermal spikes generated during transient cavitation. This may lead to the premature cleavage of the curcuminoid structure, diminishing its ability to generate ROS [78]. At 3.5 W/cm^2^, the energy is sufficient to activate it but insufficient to break the molecule. In lipid nanocarriers, the lipid bilayer acts as a shield, protecting curcumin from the direct mechanical effects of ultrasound.

Curcumin has been observed to exhibit dual activity, serving as both a pro-oxidant (for antimicrobial and anticancer effects) and an antioxidant (for wound healing, anti-inflammatory, and skin regeneration) [68, 74]. This dual activity has been found to depend on different factors [74–80]: 1) The chemical structure of curcumin (keto-enolic tautomerism); 2) curcumin concentration; 3) curcumin irradiation; and 4) the solvent. Furthermore, dual oxidant activity depends on curcumin’s location within the membrane in both eukaryotic and prokaryotic cells, as it has been observed that the molecule can order and/or disorder the lipid bilayer, thereby protecting the membrane from the entry of oxidants and peptides. Still, it can also facilitate water penetration, thereby allowing the entry of free radicals or cations and promoting oxidative stress [68, 74].

The enol form of curcumin is planar and is more stable than the keto form [80]. Both in solid phase and in most non-polar protic and aprotic organic solvents, the predominant form is enol [53, 80]. The enol tautomer of curcumin has 5 C–C bonds that can rotate freely, while the keto tautomer has 6 bonds with free rotation. This conformational flexibility enables it to bind to a wide variety of residues, allowing it to adopt the most suitable conformation to maximize hydrogen-bonding interactions, which could explain its antioxidant activity [53, 74]. In non-polar environments, curcumin exists as a closed cis-enol tautomer, characterized by an intramolecular hydrogen bond. In contrast, in polar solvents (such as physiological media), it adopts an open conformation with an intermolecular hydrogen bond, thereby enabling interaction with water molecules and polar protein groups [53, 80]. Furthermore, under special conditions, such as low pH, the keto form predominates in equilibrium with the enol form [53, 54]. Thus, it has been observed that in acidic polar solvents, curcumin exhibits antioxidant activity without the molecule being degraded, while in alkaline conditions and non-polar media, the enol form is susceptible to degradation via hydrolysis or radicalization, exhibiting pro-oxidant activity [54], further accentuated by the presence of irradiation (light or ultrasound) and whose degradation products (vanillin, ferulic acid, etc.) have been widely reported [53, 54, 81, 82]. Additionally, if the concentration is low (< 10 µg/mL), the antioxidant activity predominates [74, 83].

A study by Comeau *et al*. [83] showed that aqueous solutions of curcumin starting at 20 µg/mL generate ROS proportional to the concentration and the amount of light administered to the Gram-positive cariogenic strain *Streptococcus mutans,* regardless of the size of the curcumin granules. These results align with those obtained in this project: an increase in MRSA inhibition was observed with the aqueous dispersion of curcumin; however, the activity of the CUWSL formulation was less effective against this strain. Another study by Haukvik *et al*. [84] employed various aqueous media containing curcumin to photokill strains of *Enterococcus faecalis, Streptococcus intermedius*, and *Escherichia coli*, including liposomal formulations. It was found that the phototoxic effect of curcumin encapsulated in liposomes was limited in all strains, whether Gram-positive or Gram-negative.

As mentioned, neither bacteria nor MRSA nor *P. aeruginosa* were susceptible to PT; therefore, some reports have suggested combining PT with chelating molecules that modify the outer membrane [14]. On the other hand, in this study, *P. aeruginosa* was more sensitive to curcumin when formulated in lipid nanocarriers than MRSA. This can be explained by curcumin’s ability to form coordination compounds with cations electrostatically bound to the outer layer of lipopolysaccharides in Gram-negative bacteria, thereby forming an effective barrier against lipophilic compounds. Thus, curcumin can bind to divalent cations, thereby destabilizing lipopolysaccharides and facilitating its penetration into the bacterial membrane. This was enabled by the observed improvement with the CUWSL formulation via PT, which generated ROS. Moreover, curcumin can inhibit the Filament-Forming Temperature-Sensitive protein Z, which is essential for cell division [79]. Nevertheless, it is also worth noting the poor quantum efficiency of curcumin ROS generation, which is 4%, minimal if compared to molecules such as Rose Bengal, whose quantum efficiency is 74%, in addition to the fact that ^1^O_2_ interacts with protic polar media, attenuating the curcumin quantum efficiency [74, 85]. The primary issue with poor MRSA inhibition lies in the composition of the bacterial membrane, having a thicker cell wall. Additionally, it is essential to consider that ROS generated in PT and ST preferentially act on membranes, causing damage once oxidative stress is reached by affecting lipids, proteins, and DNA [75, 79]. For ROS to be generated, contact between the molecule and the medium-membrane interface is necessary.

Compared with Gram-positive bacteria, Gram-negative bacteria can internalize components of the medium into the periplasmic space and ultimately into the cell, facilitated by porins. Porins are proteins that form channels through the membrane and have a β-folded barrel-shaped conformation. Therefore, part of the matrix that formed nanocarriers can enter via porins. However, *P. aeruginosa* has several resistance mechanisms (mainly efflux pumps) and virulence factors (pyocyanin, biofilm formation, the SOS response, Quorum Sensing, etc.) [75, 79] that can prevent ST from being effective, as observed in PT trials. Nevertheless, polyphenols (such as curcumin) and poloxamers are molecules capable of inactivating and/or blocking efflux pumps, and their use, coupled with an increase in the quantum efficiency of ROS generation via acoustic cavitation, may explain the success of ST using curcumin against *P. aeruginosa* [14, 85, 86].

The CUWSL formulation was used not only to increase ST efficiency by preventing curcumin aggregation and protecting it, but also to maintain curcumin in its enol form within the lipid matrix (a non-polar, aprotic medium), thereby enabling activation by ultrasound radiation. Moreover, with respect to wound regeneration, lipid nanocarriers alone enhance skin function by regulating the enzymatic balance, increasing hydration, and improving collagen structure. Some poloxamers, in addition to being efflux pump inhibitors, have been found to help skin regeneration [87, 88]. Although curcumin is degraded, compounds such as vanillin and ferulic acid contribute to skin regeneration by exerting anti-inflammatory and antioxidant effects [74, 80]. Therefore, this formulation can be used in preventive therapies and in the treatment of *P. aeruginosa* skin infections, a high-priority pathogen on the WHO Bacterial Priority Pathogens List [89].

### Cytotoxicity Assays

**Fig. 11:**
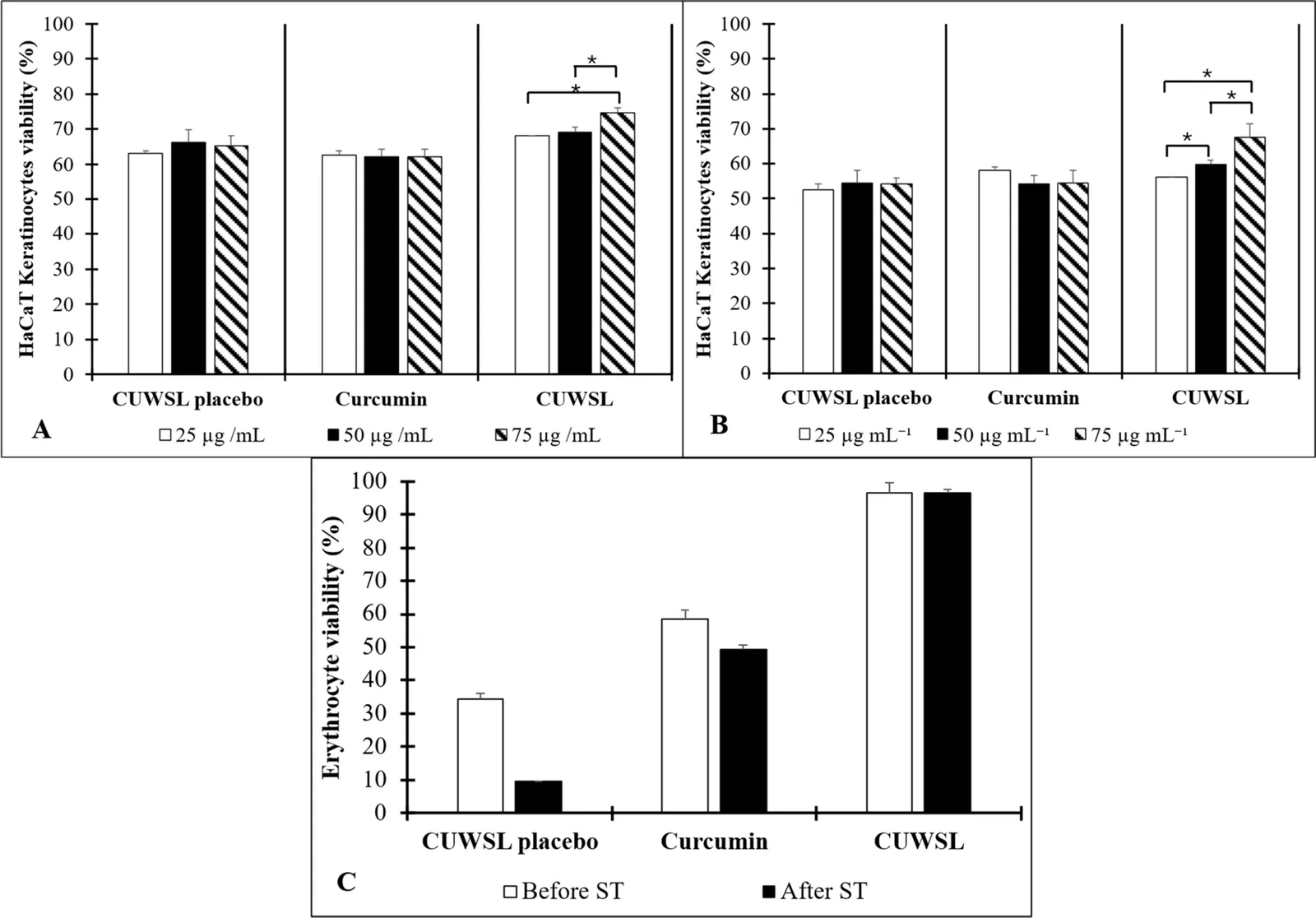
Cell viability assays for CUWSL placebo, CUWSL formulation, and curcumin in DMSO solution. (**A**) With HaCaT keratinocytes without PT, (**B**) with HaCaT keratinocytes applying PT, and (**C**) with erythrocytes with and without ST application. Error bars represent the standard deviation for n = 6 (* *p* < 0.05).

The cytotoxicity of the studied systems was evaluated in keratinocytes (HaCaT). The results are presented in Fig. 11. As shown, in the absence of PT or ST, the CUWSL placebo reduced cell viability by approximately 37%, with no significant differences across concentrations (Fig. 11A). The systems containing curcumin in DMSO showed 62% viability, with no significant differences across concentrations, whereas the CUWSL formulation exhibited increasing viability with increasing concentration, from 62% at 25 µg/mL to 74% at 75 µg/mL. When PT was applied (Fig. 11B), all systems showed decreased viability relative to the untreated systems, with no significant difference observed between concentrations or between the CUWSL placebo and curcumin-in-DMSO systems. Interestingly, the CUWSL formulation increased keratinocyte viability, both with and without light exposure, with a maximum at 75 µg/mL. Although curcumin toxicity has been studied extensively in different skin cells (fibroblasts and keratinocytes) at varying concentrations, it has been observed that at concentrations of 20 µg/mL and above, the cell viability of keratinocytes and fibroblasts ranges from 20 to 40% [90, 91]; whereas at concentrations below 2 µg/mL, curcumin can act as a protective agent against cytotoxic agents [92]. In this work, its inclusion within lipid nanocarriers could explain the reduced toxicity of the CUWSL formulation, although further studies are needed to confirm this protective effect [93]. Additional studies are necessary to fully elucidate the mechanism of interaction between free curcumin and curcumin-loaded lipid nanocarriers when applying PT. It can be inferred that curcumin, combined with PT, may exhibit antimicrobial activity without affecting skin cells, such as keratinocytes [74, 94–96], thereby making it a safe treatment option for skin infections. Regarding ultrasound tests, although the AUKEWEL® model AK-CS-02 (1 MHz) ultrasound device is approved for use on human skin according to the user manual, its application to keratinocytes caused problems due to the ultrasound intensity, which detached the cells from the wells. Therefore, to evaluate the cytotoxicity of the CUWSL formulation using ultrasound, erythrocytes were proposed as an alternative test [90–92, 97]. As shown in Fig. 11C, the viability was higher without applying ST, which occurred in the case of the CUWSL placebo and curcumin solution; however, an interesting result was that with the CUWSL formulation, there was no difference in viability with or without the application of ST, being greater than 90% in both cases. The CUWSL placebo markedly reduced erythrocyte viability to 9% when ST was applied. The presence of curcumin increased viability, reaching 48% with a curcumin solution in DMSO, whereas the CUWSL formulation exceeded 90%.

These results can be explained as follows: (i) Lipid nanocarrier composition. Phospholipids can become toxic depending on their concentration and composition. A study by Kobayashi *et al*. [98] showed that erythrocytes undergo lysis if the phosphatidylcholine contains polyunsaturated fatty acids. Furthermore, PF127 present in lipid nanocarriers can generate cytotoxic degradation products upon ultrasound exposure [99]. (ii) Curcumin in DMSO can cause changes in the shape and structure of erythrocytes, preceding toxicity and hemolysis, although this depends on the concentration to which they are exposed [100]. (iii) Curcumin may protect against hydroxyl radicals generated by acoustic cavitation. Nevertheless, in DMSO, curcumin’s efficacy is limited because it can precipitate upon contact with the aqueous biological medium. (iv) Lipid nanocarrier encapsulation ensures a high local concentration of curcumin at the cell-solvent interface, thereby scavenging sonochemically generated ROS before they initiate lipid peroxidation of red blood cell membranes. Various studies support the idea that lipid nanocarriers loaded with curcumin exhibit low hemolysis and minimal lysis of erythrocytes [101, 102].

## Conclusion

A stable lipid nanocarrier formulation containing curcumin was obtained. This formulation enhanced curcumin’s antioxidant properties, preserved its stability, and improved its skin penetration. For MRSA, inhibition was greater with Photodynamic Therapy (PT) than with Sonodynamic Therapy (ST), although inhibition never exceeded 18%. However, ST, when combined with curcumin-loaded lipid nanocarriers (CUWSL formulation), demonstrated high efficacy against *P. aeruginosa*, completely inhibiting its growth under certain conditions. These results are auspicious, particularly given the difficulty of eradicating this bacterium. These encouraging results lay the groundwork for more in-depth studies to verify the findings of this work further and test them with other Gram-negative bacteria.

The authors acknowledge the limitations of this work and, accordingly, propose the following perspectives: (i) The use of a receptor medium for release and permeation studies that increases curcumin solubility and maintains sink conditions, while simulating physiological conditions. (ii) Evaluating cytotoxicity by applying sonodynamic therapy with cells in suspension or by using substrates that reinforce cell adhesion and prevent detachment with the application of ultrasound. (iii) Carry out histological evaluation of the tissues after treatment with the CUWSL formulation, applying photodynamic and sonodynamic therapies, to determine possible tissue alterations. (iv) Characterizing the nanocarriers through cryo-TEM or SAXS to confirm their structural integrity.

## Supplementary Information

Below is the link to the electronic supplementary material.

ESM 1 (DOCX 2.02 MB)

## Data Availability

The datasets generated and/or analyzed during the current study are available as Supplementary Material or upon request.
