# Supplementary material for: Evaluating the Antimicrobial Activity of Curcumin-loaded Lipid Nanocarriers When Applying Photodynamic and Sonodynamic Therapy for the Treatment of Skin Infections
Source: Pharm Res. 2026 Oct 7;43(10):3779–802. doi: 10.1007/s11095-026-04138-9 (PMC13736448; doi:10.1007/s11095-026-04138-9)
Supplement: Supplementary file 1 [file 11095_2026_4138_MOESM1_ESM.docx]

**Supplementary material**

**Related article**

**Evaluating the antimicrobial activity of curcumin-loaded lipid nanocarriers when applying photodynamic and sonodynamic therapy**

**Authors**

Omar Molina-Alejandre ^a^, Angélica Villanueva-Martínez ^a^, María Guadalupe Nava-Arzaluz ^a^, Laura Abril Pérez-Carranza ^b^, Andrea Becerril-Osnaya ^b^, Gonzalo Hedain López-Mera ^c^, Raúl Dalí Cruz-Morales ^c^, Alma Alejandra Luna-Gómez^c^, Doris Cerecedo ^d^, Ivette Martínez-Vieyra ^d^, María Andrea Trejo-Márquez ^e^, Selene Pascual-Bustamante ^e^, Yolanda I. Chirino ^f^, Octavio Ispanixtlahuatl-Meraz ^f^, Adriana Ganem-Rondero ^a*^

**Affiliations**

^a^ Laboratorio de Investigación y Posgrado en Tecnología Farmacéutica (L-322), Facultad de Estudios Superiores Cuautitlán, Universidad Nacional Autónoma de México, Av. 1o de Mayo s/n, Cuautitlán Izcalli, C.P. 54740, Estado de México, México

^b^Bacteriología diagnóstica, Facultad de Estudios Superiores Cuautitlán, Universidad Nacional Autónoma de México Av. 1o de Mayo s/n, Cuautitlán Izcalli, C.P. 54740, Estado de México, México

^c^Departamento de Ingeniería, Facultad de Estudios Superiores Cuautitlán, Universidad Nacional Autónoma de México, Carr. Cuautitlán-Teoloyucan Km. 2.5, San Sebastián Xhala, 54714 Cuautitlán Izcalli, Estado de México, Mexico

^d^Laboratorio de Hematobiología, Escuela Nacional de Medicina y Homeopatía, Instituto Politécnico Nacional, Guillermo Massieu Helguera, No. 239, Fracc. “La Escalera”, Ticomán, Gustavo a. Madero, C.P. 07320, Ciudad de México, México

^e^Laboratorio de Postcosecha de Productos Vegetales, Centro	de Asimilación Tecnológica, Facultad de Estudios Superiores Cuautitlán, Universidad Nacional Autónoma de México, Av. Jiménez Cantú	s/n, San Juan Atlámica, Cuautitlán Izcalli, C.P. 54729, Estado de México, Mexico.

^f^Laboratorio 10, Unidad de Biomedicina, Facultad de Estudios Superiores Iztacala, Universidad Nacional Autónoma de México, Avenida de los Barrios Número 1, Colonia Los Reyes Iztacala, Tlalnepantla, C.P. 54090, Estado de México, México

**1. Validation of the analytical method for quantifying curcumin**

Table S1 shows the results of the validation of the analytical method for the quantification of curcumin encapsulated in lipid nanocarriers. The calibration curve ranged from 0.5118 to 4.662 µg/mL at 429 nm, and the medium used was a 1:1 (v/v) ethanol:water mixture. The tests were conducted in accordance with the validation guidelines of the Colegio Nacional de Químicos Farmacéuticos Biólogos de México [1].

| **Validation Parameter** | **Acceptance requirements** | **Results: WSL Formulation** | **Results: SSL Formulation** | **Verdict** |  |
| --- | --- | --- | --- | --- | --- |
|  |  |  |  |  |  |
| Specificity | Response due solely to the analyte of interest. | Response solely due to the analyte of interest. | Response solely due to the analyte of interest. | Pass |  |
|  |  |  |  |  |  |
| System precision | CV ≤ 1.5 % | 0.69% | | Pass |  |
| System linearity | r^2^= 0.98 | 0.9998 | | Pass |  |
|  | y= mx + b | Abs= (0.1389 ± 0.0004) ([Curcumin µg/mL]) + (0.001 ± 0.0012) | |  |  |
|  | IC (β1) does NOT include zero | (0.1381-0.1398) | |  |  |
| Method precision | CV ≤ 3.0 % | 1.36% | 0.54% | Pass |  |
| Method accuracy and repeatability | The arithmetic average of the % recovery is between 97 and 103% | 101.70% | 100.03% | Pass |  |
|  | CV´s % recovery is ≤ 3.0 % | 2.09% | 1.11% |  |  |
| Method linearity | r^2^= 0.98 | 0.9999 | 0.9999 | Pass |  |
|  | CI (β1) MUST include unit | (0.9939-1.0057) | (0.9972-1.0024) |  |  |
|  | CI (β0) MUST include zero | (-0.0172-0.0177) | (-0.0079-0.0074) |  |  |
|  | Arithmetic average of the % recovery is between 97-103% | 100.41% | 100.16% | Pass |  |
|  | CV´s% recovery is ≤ 3.0 % | 0.92% | 0.43% |  |  |
| Detection Limit | Informative | 0.0509 µg/mL | 0.0224 µg/mL | N/A |  |
| Quantification Limit | Informative | 0.1544 µg/mL | 0.0678 µg/mL |  |  |

Table S1. Results of the validation of the analytical method for the quantification of curcumin using ethanol:water (1:1 v/v) as solvent. WSL = Water-dispersible lecithin, SSL = Standard lecithin.

Figure S1. Results of the validation of the analytical method for the quantification of curcumin using ethanol:water (1:1 v/v) as solvent. (A) Specificity of the analytical method, (B) linearity of the system (only curcumin), (C) linearity of the method (curcumin + CUWSL placebo) and (D) Calibration curve constructed with ethanol:water (1:1) in contact with pig ear skin.

**2. Experimental design for preparation of curcumin-loaded liposomes**

Using Statgraphics statistical software, factors significantly influencing vesicle size and polydispersity index (PI) for curcumin-loaded lipid nanocarriers were identified. The results of the statistical analysis of size are presented in Tables S2 and S3 and Figures S2 and S3.

Table S2. Factorial experimental design (2^4^) with two responses for the preparation of curcumin-loaded lipid nanocarriers. WSL = Water-dispersible lecithin, SSL = Standard lecithin, PF127 = Pluronic F-127, PP123 = Pluronic P123, PI = Polydispersity index

| Experiment | Stabilizer | Phospholipid source | Solvent | Curcumin amount (mg) | Particle size  (nm) | PI |
| --- | --- | --- | --- | --- | --- | --- |
| I | PF127 | WSL | Isopropanol | 1.0 | 77.71 | 0.267 |
| II | PP123 | WSL | Ethanol | 3.0 | 63.17 | 0.208 |
| III | PP123 | SSL | Ethanol | 1.0 | 136.8 | 0.274 |
| IV | PF127 | WSL | Isopropanol | 3.0 | 80.5 | 0.282 |
| V | PF127 | SSL | Ethanol | 1.0 | 171.3 | 0.257 |
| VI | PP123 | SSL | Isopropanol | 1.0 | 140.2 | 0.269 |
| VII | PF127 | SSL | Ethanol | 3.0 | 179.4 | 0.261 |
| VIII | PP123 | WSL | Isopropanol | 3.0 | 66.33 | 0.24 |
| IX | PP123 | WSL | Ethanol | 1.0 | 53.32 | 0.16 |
| X | PF127 | SSL | Isopropanol | 1.0 | 172.8 | 0.253 |
| XI | PF127 | SSL | Isopropanol | 3.0 | 179.3 | 0.25 |
| XII | PP123 | WSL | Isopropanol | 1.0 | 60.37 | 0.166 |
| XIII | PF127 | WSL | Ethanol | 1.0 | 58.36 | 0.198 |
| XIV | PP123 | SSL | Isopropanol | 3.0 | 149.1 | 0.273 |
| XV | PF127 | WSL | Ethanol | 3.0 | 79.39 | 0.284 |
| XVI | PP123 | SSL | Ethanol | 3.0 | 157.3 | 0.272 |

Table S3. Results of ANOVA to determine the factors affecting the vesicle size of curcumin-loaded lipid nanocarriers

| Source | *Sum of Squares* | *dF* | *Mean squares* | *F-reason* | *P-value* |
| --- | --- | --- | --- | --- | --- |
| A: Stabilizer | 1779.15 | 1 | 1779.15 | 122.98 | 0.0001 |
| B: Phospholipid source | 35203.1 | 1 | 35203.1 | 2433.31 | 0.0000 |
| C: Solvent | 35.462 | 1 | 35.462 | 2.45 | 0.1782 |
| D: Curcumin concentration | 475.676 | 1 | 475.676 | 32.88 | 0.0023 |
| AB | 306.95 | 1 | 306.95 | 21.22 | 0.0058 |
| AC | 10.5625 | 1 | 10.5625 | 0.73 | 0.4319 |
| AD | 0.632025 | 1 | 0.632025 | 0.04 | 0.8427 |
| BC | 58.599 | 1 | 58.599 | 4.05 | 0.1003 |
| BD | 0.0361 | 1 | 0.0361 | 0.00 | 0.9621 |
| CD | 62.8849 | 1 | 62.8849 | 4.35 | 0.0915 |
| Total error | 72.3359 | 5 | 14.4672 |  |  |
| Total (corr.) | 38005.4 | 15 |  |  |  |

Based on Table S3, the alternative hypothesis (at least one mean is different) was accepted, with 95% confidence, for the factors:

- Stabilizer
- Phospholipid Source (B)
- Curcumin concentration (D)
- The interaction Stabilizer-Phospholipid source (AB)

Phospholipid source and stabilizer were the factors that had the most significant impact on size. When SSL and PF127 were used, the size increased, following the trend observed when a greater amount of curcumin was added, as shown in Figure S2.

Figure S2. Pareto plot for the particle size of curcumin-loaded lipid nanocarriers

Figure S3. Main effect slopes plot for the particle size of curcumin-loaded lipid nanocarriers

Figure S3 confirms the observations of the Pareto plot, with solvent being the factor that had the least impact on particle size. Similarly, the analysis was conducted for the PI response of the experimental design.

Table S4. Results of ANOVA to determine the factors affecting the PI of curcumin-loaded lipid nanocarriers

| Source | *Sum of Squares* | *dF* | *Mean squares* | *F-reason* | *P-value* |
| --- | --- | --- | --- | --- | --- |
| A: Stabilizer | 0.00182756 | 1 | 0.00182756 | 10.77 | 0.0219 |
| B: Phospholipid source | 0.00652056 | 1 | 0.00652056 | 38.43 | 0.0016 |
| C: Solvent | 0.000280563 | 1 | 0.000280563 | 1.65 | 0.2548 |
| D: Curcumin concentration | 0.00375156 | 1 | 0.00375156 | 22.11 | 0.0053 |
| AB | 0.00581406 | 1 | 0.00581406 | 34.27 | 0.0021 |
| AC | 6.25E-8 | 1 | 6.25E-8 | 0.00 | 0.9854 |
| AD | 5.625E-7 | 1 | 5.625E-7 | 0.00 | 0.9563 |
| BC | 0.000689062 | 1 | 0.000689062 | 4.06 | 0.1000 |
| BD | 0.00357006 | 1 | 0.00357006 | 21.04 | 0.0059 |
| CD | 0.0000455625 | 1 | 0.0000455625 | 0.27 | 0.6264 |
| Total error | 0.000848312 | 5 | 0.000169662 |  |  |
| Total (corr.) | 0.0233479 | 15 |  |  |  |

Based on Table S4, the alternative hypothesis was accepted (at least one mean is different), with 95% confidence, for the factors:

- Stabilizer
- Phospholipid source
- Curcumin Concentration
- Stabilizer-Phospholipid source interaction (AB)
- Phospholipid Source-Curcumin Concentration interaction (BD)

Similarly, for the PI, it can be seen from Figure S4 that Phospholipid source, curcumin concentration, Stabilizer, and AB and BD interactions significantly influenced the PI of curcumin-loaded lipid nanocarriers

Figure S4. Pareto plot for IP of curcumin-loaded lipid nanocarriers

The curcumin concentration exhibited linear behavior with a positive slope, indicating that PI increases with curcumin concentration. In contrast, the stabilizer and phospholipid source presented linear behavior with a negative slope (Figure S5).

Figure S5. Main effect slopes plot for the PI of curcumin-loaded lipid nanocarriers

Thus, considering these factors, the best-fitting model for particle size was the following:

Particle size= 102.996 - 10.9413*Stabilizer - 46.8115*Phospholipid source + 32.6497*curcumin concentration + 4.38*Stabilizer * Phospholipid source

In the same way, the equation that describes the behavior of PI for this experiment was:

PI= 0.212904 - 0.0110614*Stabilizer - 0.0499731* Phospholipid source + 0.0916916* curcumin concentration - 0.0190625* Stabilizer * Phospholipid source +0.0894461* Phospholipid source * curcumin concentration

**3. Experimental design for MRSA and *P. aeruginosa* inhibition using Sonodynamic Therapy (ST)**

Table S5. Results of the 2^4^ factorial design to determine the best conditions for MRSA inhibition by ST.

R1 and R2 show the results of two replicates

| Experiment | Exposure time (min) | US density (mW cm^-2^) | Curcumin concentration (µg mL^-1^) | Curcumin | Inhibition  (% R1) | Inhibition  (% R2) |
| --- | --- | --- | --- | --- | --- | --- |
| A | 20 | 3.5 | 50 | Dispersion | 31.7406 | 28.7697 |
| B | 10 | 5.3 | 50 | Encapsulated | 0.6955 | 0 |
| C | 20 | 5.3 | 50 | Encapsulated | 0.5196 | 0 |
| D | 10 | 5.3 | 50 | Dispersion | 3.1118 | 1.1337 |
| E | 20 | 3.5 | 50 | Encapsulated | 0.6447 | 0 |
| F | 20 | 5.3 | 50 | Dispersion | 9.7998 | 9.4875 |
| G | 10 | 3.5 | 50 | Encapsulated | 0 | 0 |
| H | 10 | 3.5 | 50 | Dispersion | 3.1511 | 1.4194 |
| I | 10 | 3.5 | 25 | Encapsulated | 6.9170 | 6.5311 |
| J | 20 | 3.5 | 25 | Encapsulated | 6.6342 | 7.6374 |
| K | 10 | 3.5 | 25 | Dispersion | 5.4182 | 4.9990 |
| L | 10 | 5.3 | 25 | Dispersion | 5.0035 | 4.3130 |
| M | 10 | 5.3 | 25 | Encapsulated | 6.2802 | 8.2966 |
| N | 20 | 3.5 | 25 | Dispersion | 7.9940 | 6.1484 |
| O | 20 | 5.3 | 25 | Dispersion | 3.5198 | 3.3251 |
| P | 20 | 5.3 | 25 | Encapsulated | 7.6461 | 5.8792 |

Table S6. Results of the 2^4^ factorial design to determine the best conditions for *P. aeruginosa* inhibition using ST

| Experiment | Exposure time (min) | US density (mW cm^-2^) | Curcumin concentration (µg mL^-1^) | Curcumin | Inhibition  (% R1) | Inhibition  (% R2) |
| --- | --- | --- | --- | --- | --- | --- |
| A | 20 | 3.5 | 50 | Dispersion | 100 | 100 |
| B | 10 | 5.3 | 50 | Encapsulated | 26.1222 | 25.2449 |
| C | 20 | 5.3 | 50 | Encapsulated | 100 | 100 |
| D | 10 | 5.3 | 50 | Dispersion | 33.9576 | 34.2687 |
| E | 20 | 3.5 | 50 | Encapsulated | 100 | 100 |
| F | 20 | 5.3 | 50 | Dispersion | 34.3478 | 33.5143 |
| G | 10 | 3.5 | 50 | Encapsulated | 9.2074 | 11.7004 |
| H | 10 | 3.5 | 50 | Dispersion | 10.1811 | 10.6835 |
| I | 10 | 3.5 | 25 | Encapsulated | 7.2271 | 8.0687 |
| J | 20 | 3.5 | 25 | Encapsulated | 28.6528 | 27.3969 |
| K | 10 | 3.5 | 25 | Dispersion | 3.4044 | 3.2096 |
| L | 10 | 5.3 | 25 | Dispersion | 1.8852 | 1.5333 |
| M | 10 | 5.3 | 25 | Encapsulated | 14.6666 | 15.0927 |
| N | 20 | 3.5 | 25 | Dispersion | 7.7168 | 8.7430 |
| O | 20 | 5.3 | 25 | Dispersion | 3.5246 | 3.6890 |
| P | 20 | 5.3 | 25 | Encapsulated | 23.6380 | 23.7842 |

The factors that significantly influence the inhibition of MRSA and *P. aeruginosa* were determined using the statistical program Statgraphics® Centurion XVI. The results for MRSA inhibition are presented in Figure S6 and Table S5 (ANOVA is shown in Table S6), while those for *P. aeruginosa* are shown in Figure S6 and Table S7.

Table S6. Analysis of variance (ANOVA) for the factors that affect the MRSA inhibition rate

| Source | *Sum of Squares* | *dF* | *Mean squares* | *F-reason* | *P-value* |
| --- | --- | --- | --- | --- | --- |
| A: Exposure time | 164.149 | 1 | 164.149 | 9.91 | 0.0051 |
| B: US density | 75.0108 | 1 | 75.0108 | 4.53 | 0.0460 |
| C: Curcumin Concentration | 1.15117 | 1 | 1.15117 | 0.07 | 0.7948 |
| D: Curcumin | 160.441 | 1 | 160.441 | 9.68 | 0.0055 |
| AB | 77.4708 | 1 | 77.4708 | 4.68 | 0.0429 |
| AC | 154.989 | 1 | 154.989 | 9.35 | 0.0062 |
| AD | 161.975 | 1 | 161.975 | 9.77 | 0.0053 |
| BC | 33.9521 | 1 | 33.9521 | 2.05 | 0.1678 |
| BD | 80.9591 | 1 | 80.9591 | 4.89 | 0.0389 |
| CD | 324.2 | 1 | 324.2 | 19.56 | 0.0003 |
| Total error | 331.425 | 20 | 16.5713 | 331.425 | 20 |
| Total (corr.) | 1569.6 | 31 |  | 1569.6 | 31 |

According to Table S6, the alternative hypothesis (i.e., that at least one mean differs) is accepted at the 95% confidence level. In this case, most of the factors and interactions influence MRSA inhibition, except curcumin concentration and US density-curcumin concentration interaction. A similar trend was also observed in Figure S6. In the Pareto chart, all these factors pass the t level, significantly affecting the MRSA microbial inhibition rate.

Figure S6. Pareto chart for MRSA microbial inhibition rate.

For *P. aeruginosa*, the same statistical analysis was performed, with a 95% confidence level, finding that neither US density nor US density-Curcumin, US density-Curcumin concentration, or Curcumin concentration-Curcumin interactions were significant and therefore did not affect the microbial inhibition of this pathogen, as shown in Table S7 and Figure S7.

Table S7. Results of ANOVA used to determine factors that affect the *P. aeruginosa* inhibition rate

| Source | *Sum of Squares* | *dF* | *Mean squares* | *F-reason* | *P-value* |
| --- | --- | --- | --- | --- | --- |
| A: Exposure time | 10460.1 | 1 | 10460.1 | 68.22 | 0.0000 |
| B: US density | 115.987 | 1 | 115.987 | 0.76 | 0.3948 |
| C: Curcumin Concentration | 13081.3 | 1 | 13081.3 | 85.31 | 0.0000 |
| D: Curcumin | 1655.18 | 1 | 1655.18 | 10.79 | 0.0037 |
| AB | 1786.53 | 1 | 1786.53 | 11.65 | 0.0028 |
| AC | 5898.03 | 1 | 5898.03 | 38.46 | 0.0000 |
| AD | 1172.85 | 1 | 1172.85 | 7.65 | 0.0119 |
| BC | 71.1356 | 1 | 71.1356 | 0.46 | 0.5036 |
| BD | 557.055 | 1 | 557.055 | 3.63 | 0.0711 |
| CD | 0.00783702 | 1 | 0.00783702 | 0.00 | 0.9944 |
| Total error | 3066.81 | 20 | 153.34 | 3066.81 | 20 |
| Total (corr.) | 37865.3 | 31 |  | 37865.3 | 31 |

Figure S7. Pareto chart for *P. aeruginosa* microbial inhibition rate

Finally, based on the experimental results, Table S8 presents the optimal values to achieve maximum inhibition against both MRSA and *P. aeruginosa*.

Table S8. Optimal value factors for MRSA and *P. aeruginosa* microbial inhibition

| MRSA | | |
| --- | --- | --- |
| *Factor* | *Optimum* | *Inhibition rate / %* |
| Exposure time | 20.0 min | 23.4998 |
| US density | 3.5 mW / cm^2^ |  |
| Curcumin Concentration | 50 µg / mL |  |
| Curcumin | Dispersion |  |
| *P. aeruginosa* | | |
| *Factor* | *Optimum* | *Inhibition rate / %* |
| Exposure time | 20.0 min | 100 |
| US density | 3.5 mW / cm^2^ |  |
| Curcumin Concentration | 50 µg / mL |  |
| Curcumin | Encapsulated |  |

**4. Conditions to perform photodynamic therapy**

The prototype lamp developed by the Department of Engineering (FESC-UNAM), along with the conditions under which photodynamic therapy was applied, are shown in Figure S8. On the other hand, Figure S9 shows the conditions tested for the application of the ST, as well as an image of the equipment used for this purpose.

Figure S8. (A) Conditions used for microbial inhibition tests with photodynamic therapy (PT). (B) An image of the photoirradiation equipment designed by the Engineering Department (FES Cuautitlan, UNAM)

Figure S9. Sonodynamic therapy (ST) equipment for the microbial inhibition tests. (A) Factorial design showing the conditions tested to apply the ST. (B) An image showing the AUKEWEL® ultrasound equipment (model AK-CS-02, 1MHz)

Figure S10 shows curcumin solubility in different aqueous systems, using the formulation components separately, together, and with the CUWSL placebo~~.~~

Figure S10. UV/Vis wavelength scan of different aqueous systems with curcumin and maximum absorbance at 421 nm

References.

[1] Colegio Nacional de Químicos Farmacéuticos Biólogos de México, 2002. Métodos analíticos: guía de validación. <https://wsqd.danednadm.site/14221491.php>. (accessed 23 July 2024).
